# Integrated multi‐omics profiling landscape of organising pneumonia

**DOI:** 10.1002/ctm2.1782

**Published:** 2024-07-31

**Authors:** Ying Tang, Cuilin Chu, Siyuan Bu, Qin Sun, Airan Liu, Jianfeng Xie, Sen Qiao, Lingyan Huang, Hongmei Wang

**Affiliations:** ^1^ Jiangsu Provincial Key Laboratory of Critical Care Medicine Department of Critical Care Medicine Zhongda Hospital School of Medicine Southeast University Nanjing China; ^2^ Shaanxi University of Chinese Medicine Xianyang China; ^3^ Assisted Reproduction Center Northwest Women's and Children's Hospital Xi'an China; ^4^ Department of Pathological General Hospital of Ningxia Medical University Yinchuan China

**Keywords:** ferroptosis, lipid metabolism reprogramming, macrophage, organising pneumonia

## Abstract

**Background:**

Organising pneumonia (OP) is one of the most common and lethal diseases in the category of interstitial pneumonia, along with lung cancer. Reprogramming of lipid metabolism is a newly recognized hallmark of many diseases including cancer, cardiovascular disorders, as well as liver fibrosis and sclerosis. Increased levels of ceramides composed of sphingosine and fatty acid, are implicated in the development of both acute and chronic lung diseases. However, their pathophysiological significance in OP is unclear. The aim of this study was to investigate the role of lipid metabolism reprogramming in OP, focusing on inflammation and fibrosis.

**Methods:**

Comprehensive multi‐omics profiling approaches, including single‐cell RNA sequencing, Visium CytAssist spatial transcriptomics, proteomics, metabolomics and mass spectrometry, were employed to analyze the tissues. OP mice model was utilized and molecular mechanisms were investigated in macrophages.

**Results:**

The results revealed a significant association between OP and lipid metabolism reprogramming, characterized by an abnormal expression of several genes related to lipid metabolism, including *CD36*, *SCD1*, and *CES1* mainly in macrophages. CD36 deficiency in alveolar macrophages, led to an increased expression of C16/24 ceramides that accumulated in mitochondria, resulting in mitophagy or mitochondrial dysfunction. The number of alveolar macrophages in OP was significantly reduced, which was probably due to the ferroptosis signaling pathway involving GSH/SLC3A2/GPX4 through CD36 downregulation in OP. Furthermore, macrophage secretion of DPP7 and FABP4 influenced epithelial cell fibrosis.

**Conclusions:**

CD36 inhibited the ferroptosis pathway involving SLC3A2/GPX4 in alveolar macrophages of OP tissue by regulating lipid metabolism, thus representing a new anti‐ferroptosis and anti‐fibrosis effect of CD36 mediated, at least in part, by ceramides.

**Highlights:**

Our findings reveal a significant association between organising pneumonia and lipid metabolism reprogramming and will make a substantial contribution to the understanding of the mechanism of organising pneumonia in patients.

## Graphical abstract

- The results revealed a significant association between OP and lipid metabolism reprogramming, characterised by an abnormal expression of several genes related to lipid metabolism, including CD36, SCD1 and CES1 mainly in macrophages.
- The number of alveolar macrophages in OP was significantly reduced, which was probably due to the ferroptosis signalling pathway involving GSH/SLC3A2/GPX4 through CD36 downregulation in OP.
- CD36 inhibited the ferroptosis pathway involving SLC3A2/GPX4 in alveolar macrophages of OP tissue by regulating lipid metabolism, thus representing a new anti‐ferroptosis and anti‐fibrosis effect of CD36 mediated, at least in part, by ceramides.

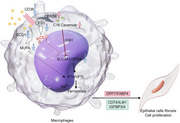

## 1 INTRODUCTION

Organising pneumonia (OP), formerly known as bronchiolitis obliterans OP, is a pulmonary condition classified as an interstitial lung disease^1^, ^2^ characterised by inflammation of the bronchioles, the alveolar connective tissue^3^ and the adjacent lung tissue. This condition can arise as a secondary manifestation of various factors, such as infection, drug toxicity, connective tissue disorders, inhalation injuries, organ transplantation and radiotherapy^4^ or it can be idiopathic. The incidence of OP is approximately 8% in patients with pneumonia. The incidence of OP is difficult to estimate because it is caused by different insults, but several studies suggest that its prevalence may exceed ∼8% of the expected level.^5^ Extensive research elucidating the pathogenesis of OP revealed a common association with lung injury that is induced by an inflammatory response. Subsequently, a cascade of tissue regeneration leads to the distinctive formation of granulation tissue within the distal lung compartments, characterising the healing process. OP disorders exhibit characteristic imaging patterns, such as irregular air‐space condensation, diffuse ground‐glass opacities, focal nodular densities and the distinctive ‘reverse halo’ phenomenon. Although corticosteroids are the primary therapeutic option, the optimal dosage and treatment duration are still under investigation. Recent investigations have identified secondary OP in some COVID‐19 patients, thus stimulating the ongoing research to understand this association to consequently formulate effective treatment plans. Investigations into the genetic and molecular mechanisms underlying OP have the potential to uncover new therapeutic targets. Despite these advancements, OP presents a research challenge due to its low frequency and the lack of large‐scale clinical trials. Further exploration of the molecular mechanisms regulating OP is needed to facilitate the identification of additional therapeutic targets that may be beneficial in patients with this condition.

Metabolism is a complex interplay of biochemical reactions that transform nutrients into metabolites of reduced size. This conversion and the resulting metabolites facilitate the production of energy, redox equivalents and macromolecules such as proteins, lipids, DNA and RNA, which are essential for cell survival and function. In most lung cells, energy expenditure is primarily devoted to perform routine cellular functions, such as mRNA transcription and protein translation. Nevertheless, specific subpopulations of cells have distinct and specialised energy‐consuming behaviours. Despite the association between cellular metabolism and various extrapulmonary diseases is known, the role of metabolic dysfunction in the development of respiratory pathology has been recently acknowledged.^6^ Nevertheless, the metabolic profile of OP remains enigmatic.

The innate and adaptive immune systems defend the host against diseases through different mechanisms, thus facilitating the emergence of simultaneous immunotherapies against OP.^7^ Innate immunity relies on a diverse array of immune cells, such as macrophages, neutrophils, monocytes, eosinophils, basophils and natural killer (NK) cells, which collectively safeguard the body's equilibrium through their inherent defensive capabilities. The function of these immune cells influences the progression of OP, as revealed by the typical severe OP that develops after COVID‐19 in an immunocompromised teenager.^8^ Immune cells possess the ability to perceive diverse signals in the microenvironment and subsequently activate specific immune functions. A correlation exists between the immune response and significant alterations in tissue metabolism, such as nutrient depletion, increased oxygen consumption and the production of reactive nitrogen and oxygen intermediates. Consequently, metabolic interventions may enhance the efficacy of immunotherapies. Nevertheless, the precise mechanism involved in metabolic reprogramming and OP immune response remains unclear. Therefore, the improvement of the understanding of the immunological microenvironment of OP would provide new perspectives on the management of this disease, potentially accelerating its eradication. Thus, this investigation addressed this gap using lung samples from patients with OP which were subjected to single‐cell RNA sequencing (scRNA‐seq) and Visium CytAssist Spatial Gene Expression analysis (10× Genomics) to explore the immunological characteristics of OP.

Alveolar macrophages are the first line of defense in the lung, contributing to the maintenance of the immune homoeostasis of the lung. Macrophages are characterised by heterogeneity and plasticity, giving them the ability to polarise into two distinct phenotypes in response to different biological and pathological conditions^9^: pro‐inflammatory and anti‐inflammatory phenotype. Recent research has transformed our understanding of how cellular metabolism influences macrophage functions.^10^ During chronic lung diseases, there was an observation of mitochondria dysfunction leading to changes in the cellular metabolic profiles of alveolar macrophages.^11^ Moreover, alveolar macrophages undergo metabolic reprogramming to generate immune responses against pathogens.^12^ This investigation assessed the impact of mitochondria on macrophage functions, and discovered mitochondrial dysfunction during chronic lung diseases. Finally, the importance of targeting metabolic pathways in alveolar macrophages was emphasised, since it may shed light on the development of new strategies against chronic lung diseases.^11^, ^13^

## 2 METHODS

### 2.1 Preparation of single‐cell suspensions

The study received approval from the ethical clearance certificate of the Ningxia Medical University General Hospital Research Ethics Committee (under protocol 2022‐66). Informed consent for sampling and subsequent analysis was provided by all patients. Our study involved patients with OP (Table S1). The computed tomography (CT) results revealed a soft tissue density shadow in the upper lobe of the right lung, accompanied by a cavity measuring approximately 4.2 × 2.7 cm^2^. This cavity exhibited an irregular shape, surrounding burr and pleural retraction, along with calcification and exudation in the vicinity of the lesion. The enhanced scan demonstrated an uneven enhancement, indicating a potential malignant tumour. The assessment of the tumour markers, cytokeratin‐19 fragment (CYFRA21‐1), neuron‐specific enolase and carcinoembryonic antigen, was negative, with values of 1.71, 6.75 and 1.39 ng/mL, respectively. However, the pathological analysis during the surgical procedure revealed a compromised inherent structure of the local lung tissue, with an evident accumulation of inflammatory cells, formation of abscesses, cystic alterations and the presence of fibroblast thrombus within the alveolar cavity, which are indicative of OP.^14^ In addition, a sample of the lung of one healthy control (HC) who was not pregnant and without tuberculosis or other lung diseases were collected.

Freshly obtained resected OP samples were washed using Hanks’ balanced salt solution (HBSS), then underwent cutting into smaller pieces, placed in a tube containing collagenase I/IV in HBSS, followed by a 30‐min incubation at 37°C under manual shaking every 10 min. The tissues were then filtered through a 70‐µm nylon mesh. Following centrifugation of the cell suspension at 400 *g* for 5 min at 4°C, the supernatant was carefully removed, and the cells in the pellet were suspended in red blood lysis buffer and then washed with HBSS before being resuspended in phosphate‐buffered saline (PBS). The cDNA library was constructed within 24 h.

### 2.2 cRNA‐seq

The .4% trypan blue dye was added to the single‐cell suspension at a ratio of 9:1. Cell counting was conducted using a Countstar Rigel S2. The percentage of living cells was determined, aiming for a minimum of 90% for quality assurance purposes.. Libraries were conducted following the manufacturer's protocol (10× Genomics, CG000086_RevM). The quality check was performed and the pooled single‐cell RNA‐seq libraries underwent sequencing on a NovaSeq 6000 platform. De‐multiplexing of samples, barcode processing and single‐cell 5′ unique molecular identifier (UMI) counting were carried out using the Cell Ranger Software Suite (v.6.0.1). Cell barcodes were automatically determined based on the distribution of UMI counts. Each cell had to meet specific criteria: gene number between 200 and 7000, mitochondrial gene percentage <.1 and haemoglobin gene percentage <.03. After filtering, a total of 30 770 cells remained for further investigation. Finally, a gene‐barcode matrix of all samples was integrated with Seurat 4.0.2. The first 50 dimensions of principal component analysis (PCA) were utilised. Harmony was used to correct actual batch effect.

### 2.3 Visium CytAssist Spatial Gene Expression

The workflow utilised for histology involved the Visium CytAssist Spatial Gene Expression technique for formalin‐fixed and paraffin‐embedded (FFPE) tissue sample. Next, 5‐µm‐thick cryosections were cut, mounted onto testing chips, positioned on a Thermocycler Adaptor with the active surface upwards and subjected to a 3‐h incubation at 42°C. Subsequently, the sections were left drying overnight, dewaxed and stained with haematoxylin and eosin (H&E) (eosin, Millipore Sigma HT110116; haematoxylin, Dako S330930‐2). Images under brightfield images were captured through a Leica DMI8 whole‐slide scanner at a 10× resolution.

The utilisation of Visium CytAssist Spatial Gene Expression for FFPE kits (10× Genomics, PN‐1000520) enabled the implementation of the spatial gene expression analysis. The tissue slices were crosslinked, followed by probe hybridisation and connection. Following the probe connection, enzymatic digestion of the RNA in the tissue section was carried out using RNase along with tissue removal enzyme (10× Genomics, PN‐3000387) to liberate the probes from the cells. These released probes were captured by the capture sequences on the Visium CytAssist Spatial Gene Expression slide using the Visium CytAssist instrument and were subsequently extended and eluted. Subsequently, the eluted probes were relocated into fresh tubes for further use.

The transfer of analytes from the tissue section to a Visium CytAssist Spatial Gene Expression slide with a .42 cm^2^ captured area was conducted by processing the glass slide using a Visium CytAssist instrument. Next, the extension of probe and construction of library were performed according to the standard Visium for FFPE workflow. Sequencing of the libraries was processed using a paired‐end dual‐indexing approach (28 cycles of read 1, 10 cycles of i7, 10 cycles of i5 and 50 cycles of read 2) on an Illumina Novaseq6000 sequencer with a minimum sequencing depth of 100 000 reads per spot using a pair‐end 150 bp (PE150) reading strategy. This sequencing process was performed by CapitalBio Technology in Beijing.

The software Spaceranger was obtained from https://support.10xgenomics.com/spatial‐gene‐expression/software/downloads/latest. Alignment, filtering, barcode and UMI counting were conducted using the Spaceranger count module to produce a feature‐barcode matrix and clusters determination. Reduction of dimensionality was achieved through PCA, and subsequent cluster generation was based on the first 10 principal components through both the K‐means algorithm and the graph‐based algorithm.

### 2.4 Proteomics

Protein expression was assessed in three OP tissues and in an equal number of control tissues. The cells were rinsed with PBS and ground in liquid nitrogen using a pestle until becoming a powder. Subsequently, the cells underwent lysis in a cold Radio Immunoprecipitation Assay buffer containing 50 mM Tris‒HCl (pH 7.5), .1% sodium deoxycholate, 1 mM Ethylene Diamine Tetraacetic Acid, 1% N‐octylglycoside, 150 mM NaCl and a complete protease inhibitor mixture. All crude protein extracts then underwent a 2‐h precipitation using acetone at −20°C and incubated with Dithiothreitol and alkylated using iodoacetamide. Following that procedure, the proteins subjected to precipitation were reconstituted in Triethylammonium Bicarbonate buffer. Subsequently, trypsin was mixed at a ratio of 1:50 (trypsin to protein), and the resulting solution was subjected to a 12−16 h incubation at 37°C. The digestion was terminated with the introduction of 2% trifluoroacetic acid. The digested proteins were subjected to desalting using C18 solid‐phase cartridges.

The samples were analysed using reverse‐phase high‐performance liquid chromatography in conjunction with mass spectrometry. Subsequent analysis of the mass spectra was conducted utilising MaxQuant computational platform (version 1.3.0.5) and Andromeda against the Uniprot FASTA human database. The proteomics data from mass spectrometry analysis were submitted to the ProteomeXchange Consortium (http://proteomecentral.proteomexchange.org) using the PRIDE partner repository (dataset identifier: IPX0003703009). Employing Lianshuan software, we conducted comprehensive bioinformatics investigations, drawing on Kyoto Encyclopedia of Genes and Genomes (KEGG) pathways for pathway insights and leveraging Gene Ontology (GO) annotations for biological process (BP), molecular function (MF) and cellular component (CC) categories. Protein quantified in a minimum of three repeats and showed at least a 2.0‐fold differential expression between groups were filtered to ensure accuracy. We normalised the dataset using *z*‐score transformation prior to engaging in an unsupervised hierarchical clustering analysis. To rigorously evaluate the statistical significance of each annotation term, a two‐tailed Wilcoxon‒Mann‒Whitney test was conducted, which was subsequently corrected for multiple comparisons with a stringent Benjamini‒Hochberg false discovery rate threshold of .05. An annotation term was deemed ‘different’ if its central tendency significantly deviated from the overall distribution. This differential analysis illuminated those terms contributing to the separation observed along principal component one, particularly between the focal condition and the periphery of cellular dynamics. The resulting output highlighted the ‘difference’ metric and corresponding *p*‐values for GO annotations and KEGG pathways. The mass spectrometry‐derived proteomics data have been archived in the ProteomeXchange Consortium (accessed via iProX, dataset ID: PXD049166), ensuring transparency and accessibility for future investigations.^15^, ^16^

### 2.5 Metabolite extraction from OP tissues

Eight hundred microlitres of cold MeOH/H_2_O was added into the frozen tissue, which was subjected to homogenisation for 1 min using a pellet pestle (Sigma‒Aldrich). The samples were centrifuged at 7000 *g* for 5 min, and a new tube was used to collect the supernatant. Then, cold methanol/water (400 µL, 80:20 [v:v], −20°C) was added, and the pellet was diluted and centrifuged again. Following this, a gentle flow of N2 gas was used to dry the supernatant, which was then preserved at −80°C until dissolution for MRM analysis by Ultivo Triple Quadrupole LC/MS System (Agilent).

### 2.6 Construction of C57 mouse OP model

Intratracheal injection of hydrochloric acid (acid) was performed on C57 mice similar to that described previously, but with some modifications.^17^, ^18^, ^19^ Male C57 mice 12 weeks old were anaesthetised using an 18 G intravenous retention needle. Subsequently, ph1.69 hydrochloric acid at a dose of 1.5 mL/kg was administered into the trachea through the glottis. The C57 mice were subjected to shaking to ensure the uniform distribution of the liquid in both lungs. The same drug at the same dose was administered twice per week for a total of two administrations to induce pneumonia. After 4 weeks, the C57 mice presented cellulose‐like exudates and macrophage aggregation in the alveolar cavity, indicating the successful establishment of the OP model after 4 weeks. Macrophages and monocytes from mouse lungs were isolated using the Monocyte Isolation Kit (Miltenyi Biotec).

### 2.7 Staining of fixed cells

Alveolar macrophages were fixed using a formaldehyde‐based fixative, since alcohol fixation is not recommended. Cells were permeated with a .1% Triton X‐100 solution, although this may potentially affect the morphology of lipid droplets. The LipidSpot stain (Biotium/LipidSpot 488 Lipid Droplet Stain, 1000×/70065‐T/20‐µL) was diluted to a 1× concentration in PBS or another suitable buffer, and if necessary, the concentration of the dye was adjusted for optimal results. Cells were incubated at room temperature in the absence of light for a minimum of 10 min or longer. Fluorescence imaging was performed using the appropriate detection channel, considering the spectral properties. Pre‐imaging cleaning is not mandatory.

### 2.8 BODIPY TR series dyeing

Alveolar macrophages were extracted in conjunction with the removal of the masking slip and then rinsed with PBS. Subsequently, they were fixed with acetone for 5 min and thoroughly cleansed with PBS. Droplets of a diluted BODIPY TR ceramide (Cer) solution with 1% bovine serum albumin (BSA) were added to fully submerge the coverslip and subjected to a 30‐min incubation at 4°C. Cells were subsequently washed multiple times with PBS supplemented with 3% BSA.

### 2.9 Transmission electron microscopy

Alveolar macrophages were subjected to trypsin digestion and subsequently collected in a centrifuge tube and underwent centrifugation at 500 *g* for 5 min, followed by storage at 4°C for 24 h with the introduction of electron microscope fixative. Cells were subjected to a sequential dehydration process, embedded in epoxy resin and sliced into 60‐nm‐thick sections. These sections were stained with a solution containing .1% lead citrate and 1% uranyl acetate, followed by examination using a transmission electron microscope (HITACHI‐HT7700). Then, ImageJ parameters were used to quantitative analysis. Initially, a low‐magnification image of the entire cell in DM3 or TIFF format was imported into ImageJ for the quantification of various parameters. Subsequently, the cell was divided into quadrants using a specific ImageJ plugin called quadrant picking (https://imagej.nih.gov/ij/plugins/quadrant‐picking/index.html, visited on 17 June 2021). Subsequent to the cell being divided into quadrants, random selection was made to analyse two quadrants in each experimental group for thorough evaluation. Maintaining precision and reproducibility is essential to achieve accurate results, thus the analysis involved a minimum of 10 cells, with three analyses carried out on cells sourced from different individuals. In cases where variability was observed among data from different individuals, increasing the cell count to 30 per individual had been shown to effectively minimise variability. During the analysis process, measurements could be managed using the ROI manager interface. Subsequently, the required parameters could be configured (analyse > set measurements: area, min and max grey value, mean grey value, integrated density, shape descriptors, perimeter, Feret's diameter, fit ellipse).^20^

### 2.10 Cell culture

The human cell line THP‐1, mouse cell line RAW264.7, normal human bronchial epithelial cells Beas2B and mouse pulmonary epithelial cells MLE‐12 were purchased from the American Type Culture Collection. Cells were cultured in RPMI‐1640 medium, high‐glucose Dulbecco's modified Eagle medium, or F12 medium (Life Technologies), supplemented with streptomycin (100 mg/mL), penicillin (100 U/mL), accompanied by 10% foetal bovine serum (FBS, Life Technologies) and incubated at 37°C under a humidified atmosphere with 5% CO_2_. THP‐1 cells underwent differentiation into macrophages via adding para‐methoxyamphetamine (PMA) (100 ng/mL), and then treated with lipopolysaccharide (100 ng/mL) and interferon‐γ (20 ng/mL) for 48 h in the presence of PMA to induce M1 polarisation. M2 polarisation was induced by the treatment with interleukin (IL)‐13 (20 ng/mL) and IL‐4 (20 ng/mL) for 48 h. C16 Cer (d16:1/16:0) (MFCD00871748, Chemical Book, Shanghai Hongye Biotechnology) was prepared at a lipid concentration of 100 µM. Subsequently, it was diluted 1:1000 (100 nM) to stimulate THP‐1 cells for 48 h prior to cell collection for experimental procedures.

### 2.11 Cell transfection

In knockdown experiments, cell transfection was conducted using mouse siCD36 (sense: 5′‐CUGAGUAGGUUUUUCUCUU‐3′; anti‐sense: 5′‐AAGAGAAAAACCUACUCAG‐3′) or transfected with human CD36‐siRNAs (sense: 5′‐UCCUCCUGGUAUUGGGAUUTT‐3′; anti‐sense: 5′‐AAUCCCAAUACCAGGAGGATT‐3′).^21^ These siRNAs were obtained from Shanghai GenePharma Company. THP‐1 cells or RAW264.7 cells numbering 2 × 10^5^ cells per well were cultured in a six‐well plate and treated with siRNA (1‒2 µg) encapsulated using the interferin reagent (Polyplus). The transfection process utilised Lipofectamine 2000 as instructed by the manufacturer. Assessment of siRNA knockdown efficiency was carried out through Western blotting 48 h post‐transfection.

For the experiment of cell fibrosis, Beas2B and MLE‐12 cells were separately treated with 20 U/mL DPP7 factor and FABP4 factor (Jining Shiye Company) for 48 h to induce fibrotic changes in the cells.

### 2.12 Cell supernatant collection

Following transfection, THP‐1 or RAW264.7 cells were plated into a six‐well plate at a density of 10^6^ cells per well. Subsequently, following the addition of 2 mL of RPMI‐1640 medium to each well, the cells were incubated at 37°C with 5% CO_2_ for 48 h. Post‐incubation, the supernatant was harvested through centrifugation at 2000 rpm. It is crucial to uphold sterile conditions throughout the culturing procedure and routinely sanitise the incubator. Protein secretion is the most prevalent secretion of THP‐1 or RAW264.7 cells. Therefore, proteomics and enzyme‐linked immunosorbent assays (ELISAs) are employed for identification purposes.

### 2.13 RT‐PCR

Samples were subjected to Trizol solubilisation for subsequent quantitative RT‐PCR analysis, and RNA isolation was performed using a kit following the manufacturer's protocol (12183555, Invitrogen). An Evo M‐MLV reagent kit (AG11706, Accurate Biology) was used to perform the reverse transcription of RNA. Quantitative PCR was conducted utilising the ABI7500 Real‐time System (Thermo Fisher Scientific) and SYBR Green Pro Taq HS reagent kit (AG11701, Accurate Biology) to quantify cDNA levels (Table S2). The method of 2^‒ΔΔCT^ was employed to determine relative gene expression. Gene expression fold changes were determined by comparing the cDNA quantity in the treated samples with that in the calibrator sample (vehicle).

### 2.14 Western blot

The extracted total proteins were subjected to electrophoresis using Sodium Dodecyl Sulfate Polyacrylamide Gel Electrophoresis, followed by transferring onto a Polyvinylidene Fluoride (PVDF) membrane and blocked in 5% skimmed milk at room temperature for 1 h. The membrane underwent three washes with Tris Buffered Saline with Tween‐20 every 10 min. The PVDF membrane was treated with rabbit anti‐SCD1 polyclonal antibody (1:1000, 28678‐1‐AP, Proteintech Group), rabbit anti‐CD36 polyclonal antibody (1:1000, 18836‐1‐AP, Proteintech Group), rabbit anti‐CES1 polyclonal antibody (1:1000, 16912‐1‐AP, Proteintech Group), rabbit anti‐β‐actin monoclonal antibody (1:1000, 66009‐1 Ig, Proteintech Group), rabbit anti‐FTH1 monoclonal antibody (1:1000, R382071, Chengdu Zen Bioscience) or mouse anti‐FTL monoclonal antibody (1:1000, 220988, Chengdu Zen Bioscience), mouse anti‐GPX4 monoclonal antibody (1:1000, 67763‐1‐Ig, Proteintech Group), rabbit anti‐CD98/SLC3A2 polyclonal antibody (1:1000, 15193‐1‐AP, Proteintech Group), rabbit anti‐vimentin polyclonal antibody (1:1000, 10366‐1‐AP, Proteintech Group), rabbit anti‐N‐cadherin polyclonal antibody (1:1000, 22018‐1‐AP, Proteintech Group) and rabbit anti‐E‐cadherin polyclonal antibody (1:1000, 20874‐1‐AP, Proteintech Group) followed by overnight incubation at 4°C in a shaker. After rinsing, the membrane was incubated with Horseradish Peroxidase (HRP)‐labelled sheep anti‐rabbit antibody (1:5000, 111‐035‐144, Jackson ImmunoResearch Laboratories) or HRP‐labelled sheep anti‐mouse antibody (1:5000, 115‐035‐003, Jackson ImmunoResearch Laboratories) at room temperature for 90 min. Subsequently, Enhanced chemiluminescence luminescence detection was utilised to visualise the presence of protein bands.

### 2.15 DPP7/FABP4 ELISA

THP‐1 cells were stimulated to induce differentiation into M2 type and treated with siCD36 for 48 h. Following centrifugation at 3000 rpm for 10 min, the cell supernatant was collected. Subsequently, 10 µL of the centrifuged supernatant, 40 µL of sample dilution buffer and 100 µL of HRP‐labelled DPP7 or FABP4 antibody were sequentially added to each well. The sealed reaction wells were then placed in an incubator at 37°C for 60 min. Following the incubation period, the liquid was discarded, wash buffer was introduced into every well, allowed to stand for 1 min and then removed. This washing process was repeated five times. Subsequently, substrates A and B were added to each well, followed by incubation at 37°C in the absence of light. Within 15 min, 50 µL of stop solution was introduced into each well, and the OD value of each well was assessed at 450 nm.

### 2.16 Fe^2+^ ELISA

Alveolar macrophages were treated with siCD36 for 48 h, followed by centrifugation and subsequent collection in EP tubes. The cells were resuspended within a buffer solution, homogenised through sonication followed by centrifugation at 10 000 *g* for 10 min, after which the resulting supernatant was collected. Upon obtaining the supernatant, it was meticulously merged with the chromogenic agent and subjected to a 10‐min incubation period at 37°C, as per Elabscience's E‐BC‐K773‐M protocol. Following centrifugation, the supernatant was delicately aspirated into a 96‐well plate, paving the way for absorbance readings at 593 nm through a BioTek SynergyH1 microplate reader. In a subsequent experiment, 60 µL of serum was expertly combined with 180 µL of buffer solution and then spun at 5000 *g* for 5 min. This process led to the collection of 200 µL of the resultant supernatant, which was evenly distributed across the wells, accompanied by the addition of 100 µL of chromogenic agent to each well. After a subsequent 10‐min incubation at 37°C, the optical density was quantified at 593 nm using the microplate reader.

### 2.17 Glutathione ELISA

In an experimental manoeuver aimed at deciphering CD36's role in alveolar macrophage function, cells were subjected to CD36 silencing for 48 h. Following this, the harvested cells were suspended in 150 µL PBS and disrupted by sonication. The resultant suspension, combined with 100 µL of a proprietary precipitating agent (Nanjing Jiancheng Bioengineering Institute, A006‐2‐1), was centrifuged gently at 3500 rpm for a 10‐min interval. The supernatant, which was carefully collected, was dispensed into a 96‐well plate in 100 µL aliquots. To each well, buffer agent and chromogenic agent were sequentially added, allowing the mixture to equilibrate at room temperature for 5 min before assessing absorbance at 405 nm using a microplate reader, a critical step in quantifying the cellular response.

In parallel, a similar protocol was adapted for mouse serum analysis. A blend of 50 µL of serum and 200 µL of the precipitating agent was centrifuged under identical conditions. The clear supernatant was then aspirated and transferred to a separate 96‐well plate, where it underwent the same downstream procedures as the macrophage homogenates, thereby ensuring comparative consistency in the analytical approach.

### 2.18 Malondialdehyde ELISA

Following treatment with siCD36, alveolar macrophages were processed by sonication after being harvested and diluted in 250 µL of PBS. The resulting homogenate underwent centrifugation at 1000 *g*. Subsequently, the resulting supernatant was combined with reagent I, reagent II and reagent III (Nanjing Jiancheng Bioengineering Institute, A003‐2) in specific volumes. This mixture underwent a 40‐min incubation in a 95°C water bath, cooled and transferred to a microplate reader for OD value measurement at 532 nm.

### 2.19 Determination of ATP levels

Following the culturing, harvesting and quantification of alveolar macrophages, each well of a 96‐well plate was seeded with 20 000 cells. Subsequently, the cells underwent treatment with siCD36 for 48 h. CellTiter‐Glo Reagent (Promega, G7572) was introduced into each well, following which the 96‐well plate was left to incubate at room temperature for 10 min. Finally, the luminescence from the microplate reader was used to determine the ATP levels.

### 2.20 Lipid peroxidation assay

The alveolar macrophages were treated with BODIPYTM 581/591 C11 (D3861, Thermo Fisher Scientific) and subsequently incubated at 37°C for a duration of 30 min. The cells underwent a PBS wash and subjected to the analysis of reactive oxygen species (ROS) by flow cytometry (FACS Calibur, Becton, Dickinson and Company).

### 2.21 Statistical analysis

We conducted statistical analyses with GraphPad Prism 8.0.2. To assess differences between dual entities, a Student's *t*‐test was employed, whereas one‐way ANOVA, complemented with Tukey's post hoc test, was utilised for comparisons among multiple groups. The data are depicted as the mean ± SEM, with a threshold of significance set at *p* < .05.

## 3 RESULTS

### 3.1 Changes in proteomic signatures and cellular composition of OP tissue

**FIGURE 1:**
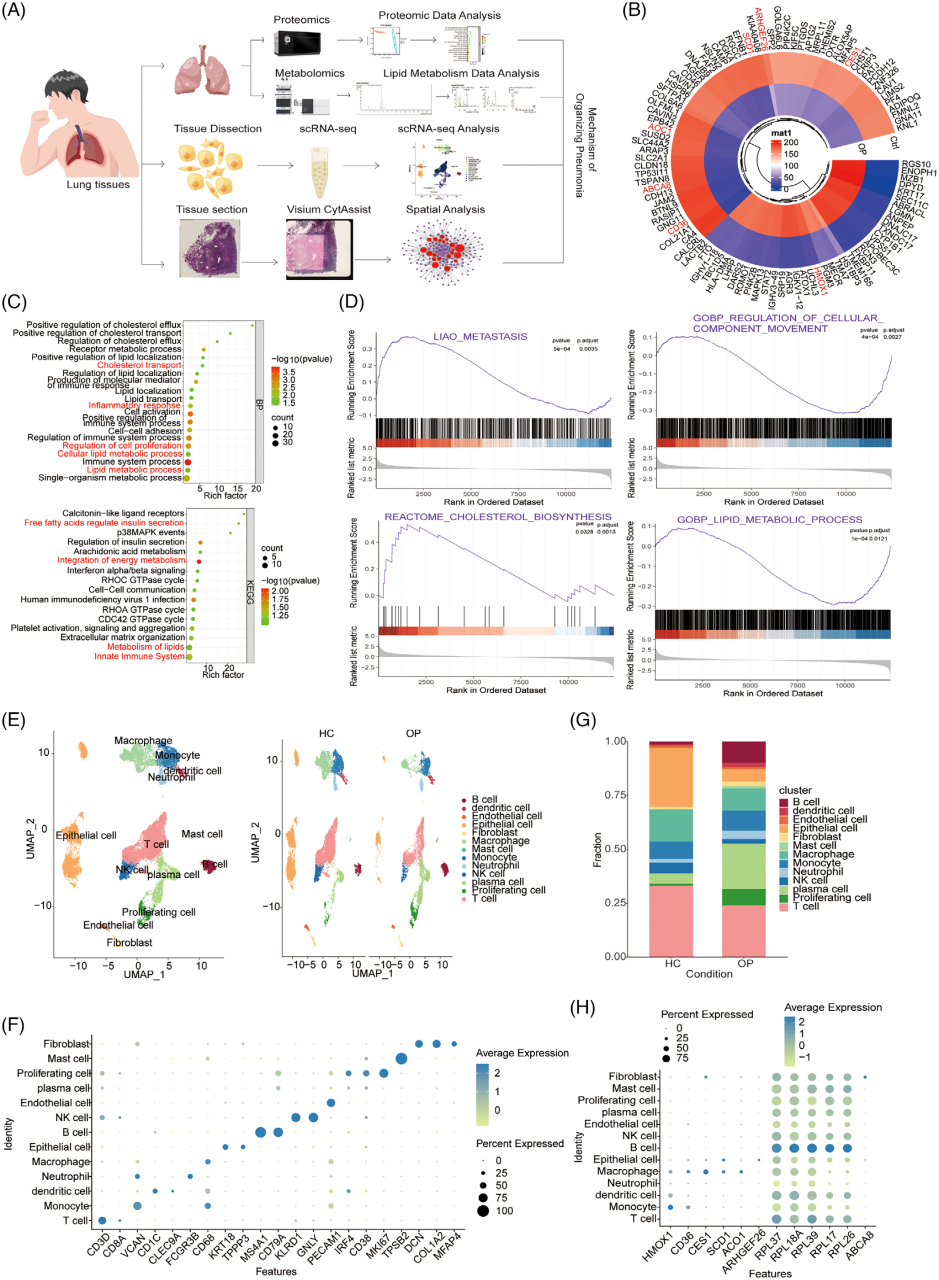
The cellular composition of organising pneumonia (OP) tissue. (A) Workflow of proteomics, metabolomics, single‐cell RNA sequencing (scRNA‐seq) and Visium CytAssist applied to lung tissues. (B) Unsupervised hierarchical clustering was performed on 116 label‐free quantitation protein intensities (log2) that were quantified in three replicates of the two groups' proteomes. The intensities were required to exhibit a fold change (FC) greater than 2.0 and a *p*‐value less than .05 in their abundance between two groups. The resulting heatmap displayed the grouped LFQ protein intensities, transformed using *z*‐scores and log2. (C) Gene Ontology (GO) enrichment analysis for the biological processes involving the upregulated and downregulated proteins and Kyoto Encyclopedia of Genes and Genomes (KEGG) analysis for the pathways changed between OP and healthy control (HC) groups. (D) Gene set enrichment analysis (GSEA) analysis revealed that the changed proteins were mainly enriched in metastasis, regulation of cellular component movement, lipid metabolic process and cholesterol biosynthesis. (E) UMAP of cells identified from the scRNA‐seq data of HC and OP tissues. (F) Expression matrix of cell‐type marker genes in the 13 cell types within OP tissues. (G) Proportion of each cell type in HC and OP samples. (H) Expression matrix of lipid metabolism genes in the 13 cell types within OP tissues. LFQ, label‐free quantification.

scRNA‐seq, spatial transcriptomics, metabolomics and proteomics were used to examine the OP tissue and learn more about the pathogenesis of OP (Figures 1A and S1). The OP tissues were examined by CT, H&E staining and immunohistochemistry. Three OP samples and three control samples were used to assess the mechanisms of OP development by proteomic analysis. A total of 3878 proteins from two distinct group, such as normal group and OP group, were quantified, with six biological replicates for each condition. The proteomic data set of 12 samples was subjected to PCA, which identified distinct clusters of replicate samples for both the HC and OP groups, indicating a similarity in protein expression within each group (Figure S2A). The expression profile of the protein read count was further analysed using volcano plots, revealing 231 upregulated proteins and 158 downregulated proteins between the OP and HC groups (fold change [FC] > 2, *p *< .05) (Figure S2B). Furthermore, a change in the expression level of the top 100 differentially expressed proteins was discovered through the hierarchical clustering analysis of proteins. A significant proportion of these differentially expressed genes (DEGs) participated in lipid metabolism, as emphasised in red. GO enrichment analysis was performed to identify BP, CC and MF to assess the resemblances and disparities between the OP and HC groups (FC > 2, *p *< .05). The findings suggested that the proteins changed in the OP group showed enrichment in pathways associated with the immune system response, lipid metabolic process, lipid localisation and regulation of cell proliferation, as demonstrated in Figures 1C and S2C,D. Furthermore the signalling pathways primarily associated with the integration of energy metabolism, innate immune system and lipid metabolism were more prevalent in the OP group compared to the HC group (Figure 1C), as revealed by the KEGG analysis. Given the limitations of proteomic analysis in the evaluation of the roles of DEGs, gene set enrichment analysis (GSEA), which is a widely used method to examine and interpret coordinated pathway alterations, was performed on all proteins. Our findings revealed an upregulation of processes such as metastasis and cholesterol biosynthesis in the OP group. Conversely, processes including the regulation of cellular component movement, lipid metabolic processes, cholesterol biosynthesis, stem cell activity and molecules associated with elastic fibers, were downregulated (Figures 1D and S2E). These proteomic analyses further confirmed that OP regulated immune system response, lipid metabolic process, lipid localisation, molecules associated with elastic fibres and cell proliferation, thus providing a supportive microenvironment for OP formation.

Lung samples were collected for scRNA‐seq from one patient with OP, one HC (GEO accession number: GSE250404) and a publicly available lung sample (GSE164829) to characterise the cellular composition of the lung tissues in these three samples (Figures 1E‒G, S1 and S3). A total of 30 770 cells passed the quality control (Figure 1E), and among them, 13 cell types representing canonical cell markers were found (Figures 1F and S3), including B cells (1303 cells), dendritic cells (358 cells), endothelial cells (323 cells), epithelial cells (6068 cells), fibroblasts (438 cells), mast cells (135 cells), macrophages (4088 cells), monocytes (2632 cells), neutrophils (736 cells), NK cells (1225 cells), plasma cells (3270 cells), proliferating cells (987 cells) and T cells (9207 cells). Endothelial cells were identified by the typical marker *PECAM1*, and they were also characterised by the markers *TPPP3* and *KRT18*, as shown in Figure S3. The two markers *DCN* and *COL1A2* were used to identify fibroblasts, while the marker *MKI67* was associated with the proliferation of OP fibrosis. In terms of immune cell typing, B cells were recognised by their high expression of *MS4A1* and *CD79A*, while CD1C and *CLEC9A* were used to identify dendritic cells. *TPSB2* was the classical marker of mast cells, while *VCAN* was used to identify monocytes. The marker *CD68* typical of macrophages was also found. *FCGR3B* was used to detect neutrophils. *KLRD1* and *GNLY* were used to distinguish NK cells. Plasma cells were identified by *CD38* and *IRF4*. T cells were identified by the specific expression of *CD3D* (Figure 1F). Notably, the proportion of B cells, plasma cells and proliferating cells increased significantly in the OP group compared to the normal group, which was probably attributed to the vaccination history of the patient. Moreover, the proportion of epithelial cells, macrophages, NK cells and T cells were obviously decreased in the OP group compared to the normal group (Figure 1G). Proteomic results showed that OP disease was mainly related to lipid metabolic reprogramming and the expression of genes related to it revealed that these genes were expressed in different cell types. Genes related to ribosomal lipid metabolism, such as *RPL* (*37*, *18A*, *39*, *17* or *26*), showed a relatively broad spectrum of expression, especially in B cells. *HMOX1* was mainly expressed in monocytes. Genes related to fatty acid metabolism, including *CD36*, *CES1*, *SCD1* and *ACO1*, were especially expressed in macrophages. *ABCA8* expression was found in fibroblasts (Figure 1H).

### 3.2 Functional characteristics of different cell types in OP

**FIGURE 2:**
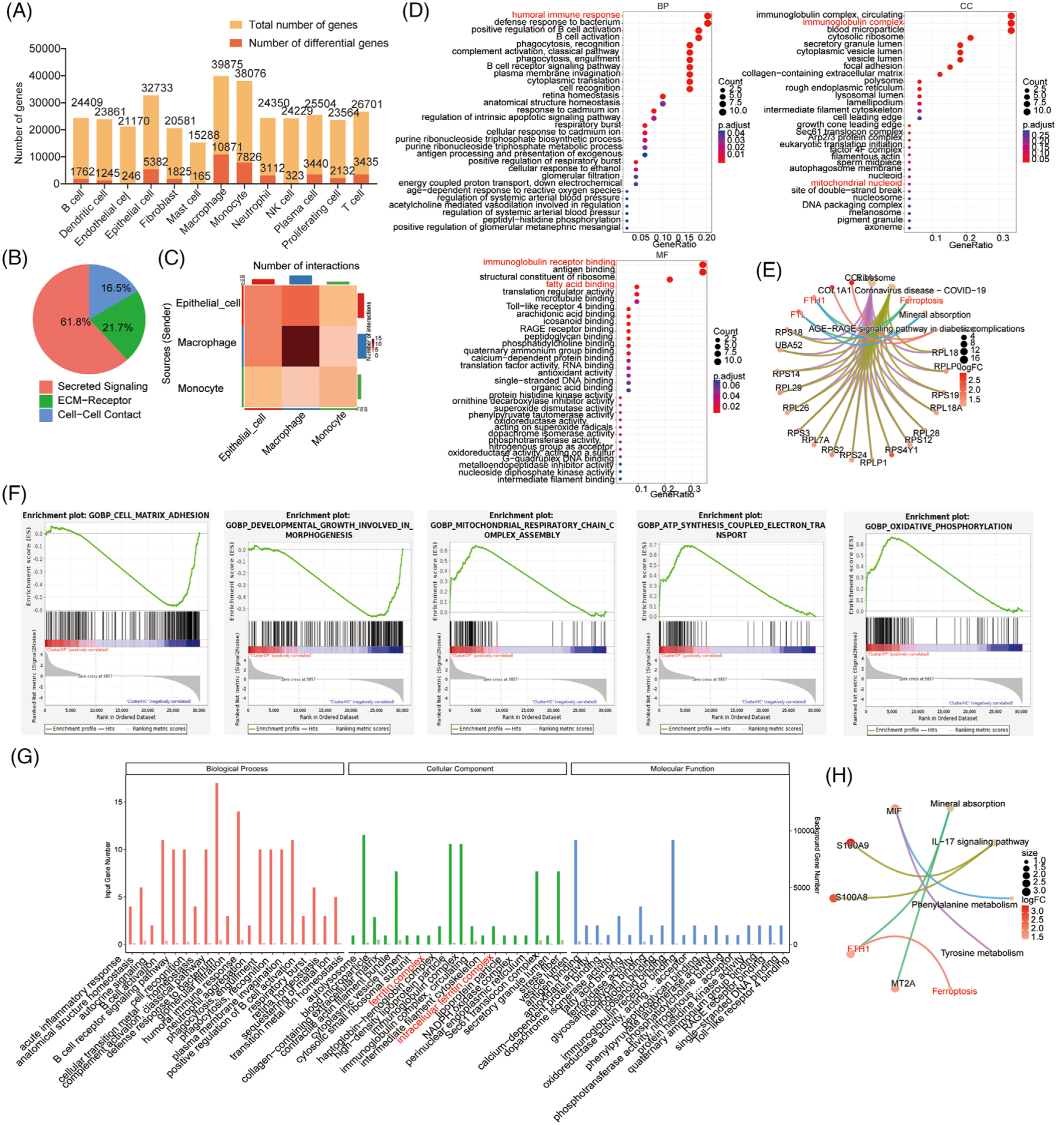
Functional analysis was performed using the differentially expressed genes (DEGs) within the related cells between organising pneumonia (OP) and healthy control (HC) tissues from the single‐cell RNA sequencing (scRNA‐seq) data. (A) The numbers of total genes and DEGs in each cell type of OP group compared to HC group. (B and C) Analysis of cell communication patterns, including secreted signalling, ECM receptor and cell‒cell contact, among monocytes, macrophages and epithelial cells. (D) Gene Ontology (GO) enrichment analysis was performed on macrophages. (E) The significant regulatory pathways of key genes were analysed by Kyoto Encyclopedia of Genes and Genomes (KEGG). (F) Gene set enrichment analysis (GSEA) revealed that the changed proteins were mainly enriched in cell‒matrix adhesion, developmental growth involved in morphogenesis, mitochondrial respiratory chain complex assembly, ATP synthesis coupled electron transport and oxidative phosphorylation in macrophages. (G) GO enrichment analysis was performed on epithelial cells. (H) The significant regulatory pathways regulated by pivotal genes were analysed by KEGG. ECM, extracellular matrixc; LFQ, label‐free quantification.

GO and KEGG enrichment analyses of DEGs in the OP and HC groups were performed using the scRNA‐seq data and the differences were compared. Defense response to bacterium, immune response, immunoglobulin complex, immunoglobulin receptor binding, lipid and atherosclerosis, and focal adhesion were the biological pathways that were enriched in DEGs in OP tissues (Figure S4A,B). Additionally, cell cycle and DNA replication were found through GSEA as also enriched in DEGs in OP tissues (Figure S4C). Furthermore, the analysis of DEGs and the total number of genes in different cell types revealed that the number of DEGs in epithelial cells, macrophages and monocytes were more than that in other cell types (Figure 2A). The DEGs in each cell type were analysed to further explore the role of the 13 different cell types in OP. The volcano plots confirmed more DEGs in epithelial cells, macrophages and monocytes than in other cell types (Figure S5). Subsequently, the assessment of the cellular communications among epithelial cells, macrophages and monocytes revealed that secreted signalling accounted for 61.8%, extracellular matrix receptor‐related pathways accounted for 21.7% and cell‒cell contact accounted for 16.5% (Figure 2B). Among these three cell types, the interaction between macrophages and epithelial cells was notably prominent (Figure 2C). Taken together, monocytes, macrophages and epithelial cells, especially the last two, play a potentially crucial role in the development of OP.

GO‒KEGG analyses on epithelial cells, macrophages and monocytes revealed that BP in monocytes mainly included lipid transport, positive regulation of proteolysis and regulation of cholesterol transport (Figure S4D). KEGG pathway of monocytes revealed that phagosome, cell adhesion molecules, lipid and atherosclerosis, and MAPK signalling pathway were involved in OP (Figure S4E). GSEA analysis also showed the involvement of monocytes in the biosynthesis of unsaturated fatty acids and primary immunodeficiency (Figure S4F). As mentioned above, the analysis on the proportion of epithelial cells and macrophages demonstrated their significant decrease in the OP group (Figure 1G). DEGs of macrophages in OP and HC groups were mainly involved in humoural immune response and defense response to bacterium, as demonstrated by BP analysis. Immunoglobulin complex by binding immunoglobulin receptor was identified as the primary function in macrophages of OP by CC and MF analyses (Figure 2D). Macrophages of OP exhibit heightened expression of ferroptosis regulators, FTH1 and FTL, and ribosome‐related transcripts (Figure 2E). GSEA further underscores their involvement in modulating extracellular matrix adhesion, developmental processes linked to morphogenesis, and mitochondrial functions such as respiratory chain complex assembly and ATP synthesis via electron transport (Figure 2F). Notably, the observed budding phenomenon mirrors a process where epithelial sheets, with robust cell‒matrix and weakened cell‒cell adhesions, undergo dynamic transformations.^22^ While these adhesive dynamics are vital for normal cell survival, cancer cells often exploit this mechanism to evade apoptosis, highlighting potential therapeutic targets.^23^ Our hypothesis was that the downregulation of cell matrix adhesion and developmental growth involved in the morphogenesis of macrophages led to epithelial cell death and morphological changes of OP. Apoptosis of lung alveolar epithelial cells (AECs) is an early pathogenic event in the development of OP, although its causative mechanism is still unclear. The number of AECs showed a cliff‐like decrease while DEGs significantly increased in OP (Figures 1G and 2A). Moreover, GO terms showed that AECs in OP were mainly involved in defense response to bacterium, humoural immune response by regulating immunoglobulin complex through the binding of the immunoglobulin receptor (Figure 2G). The ferroptosis‐related gene (*FTH1*) expression was also increased in AECs, as revealed by KEGG of DEGs (Figure 2H). The results of the functional analysis of AECs were similar to those of macrophages, which might be related to the death of AECs caused by macrophage phagocytosis.^24^

The trajectory analysis of the differentiation of the monocytes into macrophages and the epithelial cells into fibroblasts was performed, as shown in Figure S6. The pseudo‐temporal analysis of cells in OP showed that monocytes transformed into macrophages (Figure S6A). The monocle method uses the branched expression analysis modelling to analyse the pseudo‐temporal cell data and the specified nodes, identifying DEGs related to branches and allowing to focus on these genes. The trajectory analysis indicated a significant activation of pathways in which inflammatory cytokines and chemokines (*CXCL3*, *CCL2*, *CXCL2*, *HIF1A*, *CCL3*, *CXCL8*, *DUSP1* and *CCL3L1*) and vesicular transport (*EREG*, *SNX9*, *AREG*, *B2M, SFTPB*, *SFTPC*, *SFTPA2* and *SFTPA1*) are involved, during the transformation of monocytes into macrophages (Figure S6B). Additionally, the GO term analysis of the changed expression of genes during the differentiation of the monocytes into macrophages in OP showed the involvement of lipid metabolism including long‐chain fatty acid transport and negative regulation of lipid localisation, as well as inflammatory response, immune system process such as macrophage activation, regulation of epithelial cell proliferation and cytokine‐related pathway (Figure S6C). The pseudo‐temporal analysis of cells in OP showed that epithelial cells transformed into fibroblasts (Figure S6D). The trajectory analysis showed a significant activation of pathways associated with extracellular matrix organisation (*RGCC*, *CAV1*, *TIMP1*, *FBLN1*, *COL3A1*, *COL1A1*, *COL6A3*, *COL1A2*, *COL6A2*, *CCN2*, *CCN1* and *A2M*), collagen binding (*FN1*, *SPARC*, *LUM*, *DCN* and *SPARCL1*) and immune response (*CXCL2*, *GAPDH*, *CCL2*, *JCHAIN*, *IGLC3*, *IGHG4*, *IGHG3*, *RARRES2*, *IGLC2*, *IGHG1*, *IGHA1*, *IGKC* and *CFD*), during the transformation of epithelial cells into fibroblasts in OP (Figure S6E). Lipid transport, macrophage migration, macrophage chemotaxis, immune response, regulation of epithelial cell proliferation and fibroblast proliferation, as well as homeostatic processes were activated during the differentiation of the epithelial cells into fibroblasts (Figure S6F). Furthermore, in normal conditions, fibroblasts exhibit lower levels of fibrosis compared to OP, and they are positioned in the intermediate stage of differentiation (OP is positioned at the terminal stage of differentiation). This implies that under physiological conditions, this fibrosis might be reversible (Figure S7A,B). Conversely, in the context of OP, fibrosis is entirely irreversible. Significant differences were observed in the expression of DEGs between the normal group and the OP group (Figure S7C). In summary, the functions of lipid metabolism, immune response and cell proliferation are significantly upregulated during both the differentiation of monocytes into macrophages and the differentiation of epithelial cells into fibroblasts.

**FIGURE 3:**
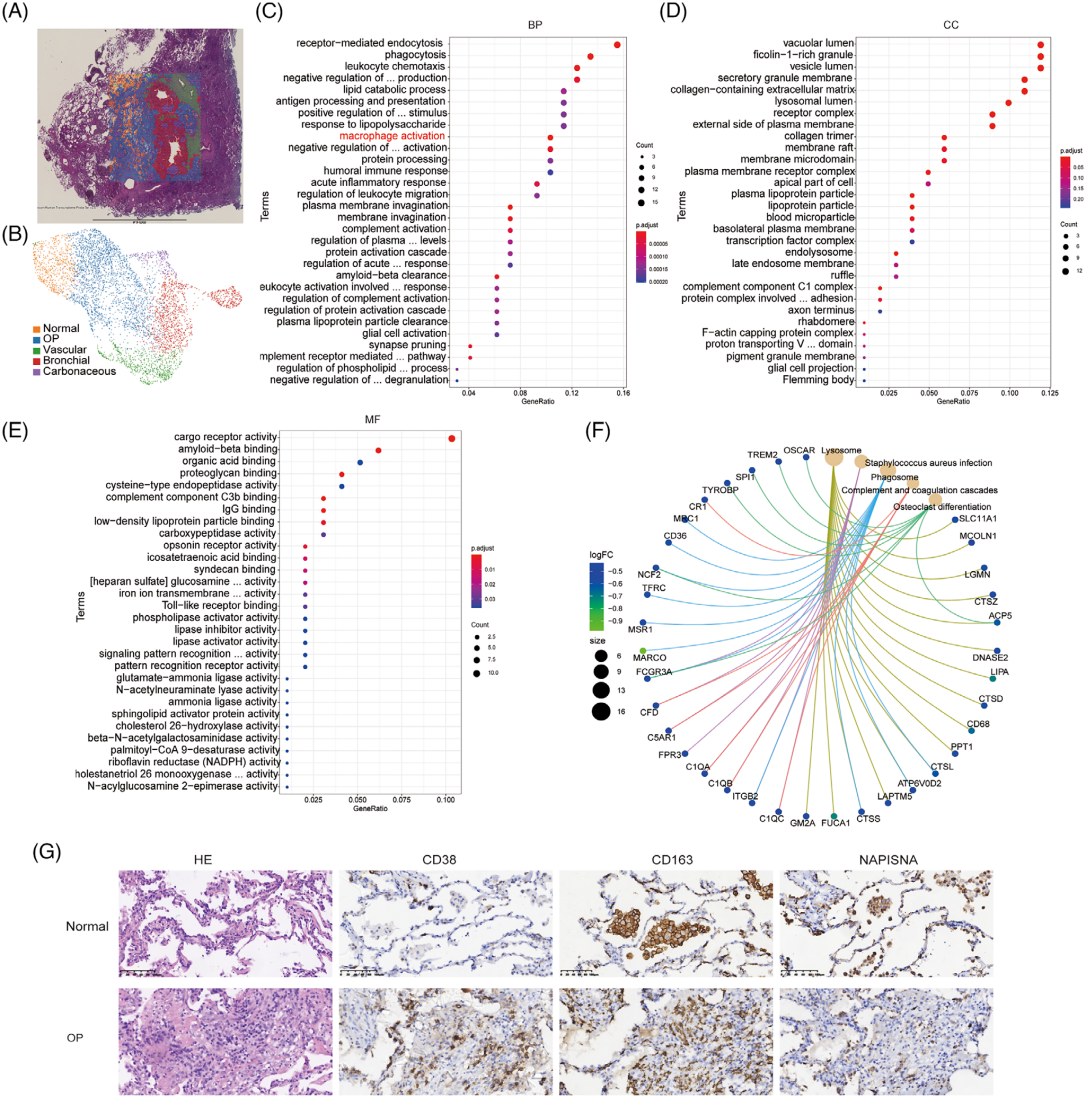
Spatial transcriptomic analysis of the immunity and energy metabolism heterogeneity in organising pneumonia (OP). (A and B) Distribution of Visium CytAssist clusters in OP samples. (C‒E) Gene Ontology (GO) enrichment of major Visium CytAssist clusters (OP/normal), including biological processes (C, BP), cellular components (D, CC) and molecular functions (E, MF). (F) Kyoto Encyclopedia of Genes and Genomes (KEGG) was used to analyse the major functional changes of OP regulated by related genes. (G) Haematoxylin and eosin (H&E) staining and immunohistochemical staining were used to analyse multiple markers (CD38, CD163 and NAPISNA) in the lungs of OP patients.

The OP tissue slices were used to perform spatial transcriptomics (GEO accession number: GSE250385). The morphology of the OP tissue was assessed by H&E staining, and included normal area, OP area, vascular area, bronchial area and carbonaceous parts. Thus, the spatial transcriptome spots were divided into five clusters according to these areas (Figures 3A,B and S8). GO term (BP) analysis showed the upregulation of receptor mediated endocytosis, phagocytosis, leukocyte chemotaxis and lipid catabolic process in the OP area (Figure 3C). Vacuolar lumen, ficolin‐1‐rich granule, vesicle lumen, secretory granule membrane and collagen‐containing extracellular matrix were involved in CC as revealed by GO analysis (Figure 3D). The above function could be related to cargo receptor activity in MF in OP (Figure 3E). KEGG analysis revealed that the functional regulation of sub‐organelles, such as phagosome and lysosome, is regulated by the genes *CD36*, *TFRC*, *MARCO* and *ATP6V0D2* associated with lipid metabolism (Figure 3F). CD38 expression was significantly increased in B and T lymphocytes in OP, while CD163‐labelled macrophages and NAPISNA‐labelled epithelial cells were significantly decreased in OP tissue, which agreed with the scRNA‐seq data in OP tissues (Figure 3G).

### 3.3 Lipid metabolic reprogramming in macrophages in OP patients

**FIGURE 4:**
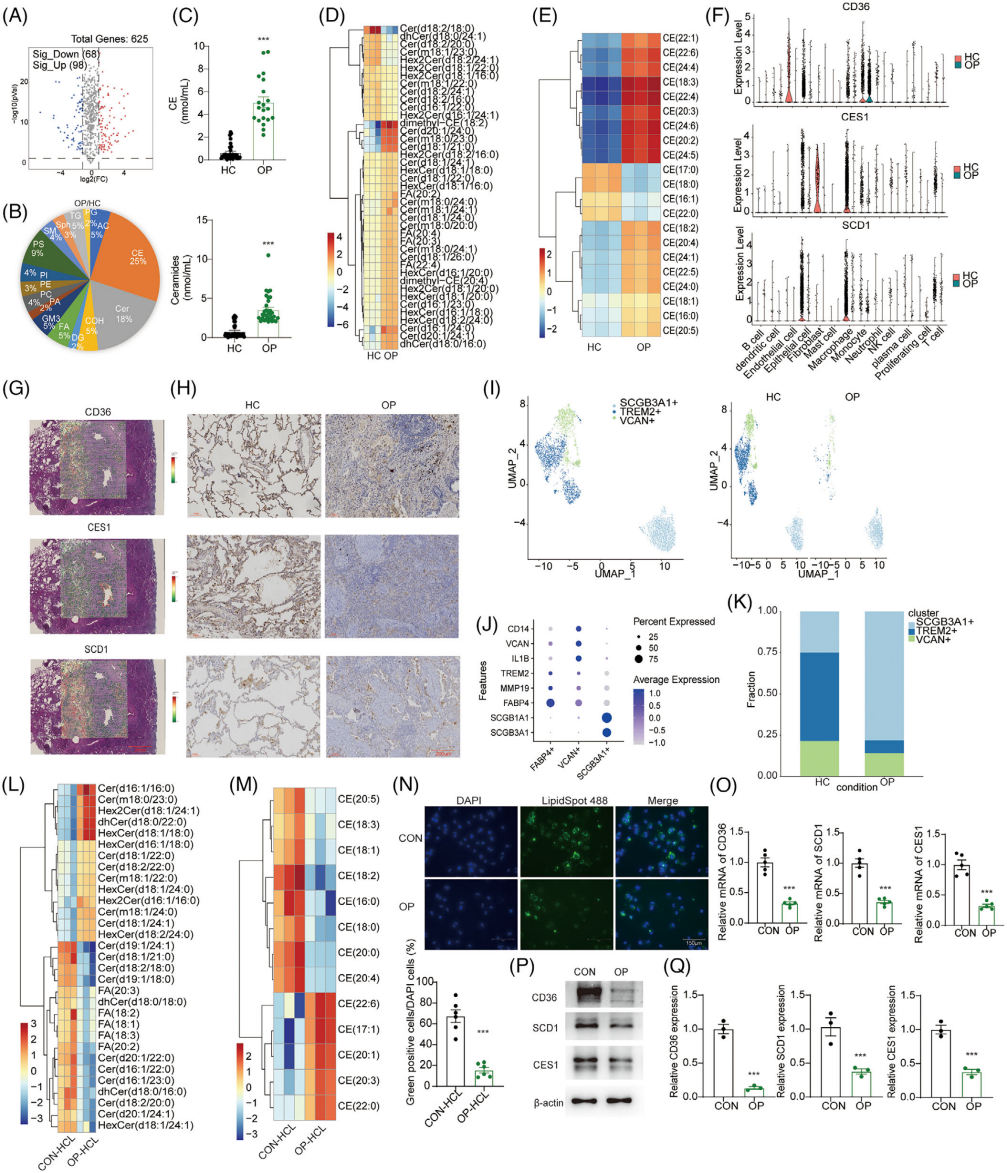
Abnormal macrophage lipid metabolism, including cholesterol ester (CE) and ceramides (Cer) in organising pneumonia (OP) patients. (A) Volcano plots revealed that 166 lipid metabolites showed significant differences, with 98 upregulated and 68 downregulated (variable important in projection >1.0 and *p*‐value <.05). (B) The percentile pie chart showed the proportion of different types of lipid metabolites. Among these, CE and Cer were the most abundant. (C) CE and Cer were significantly increased analysed by enzyme‐linked immunosorbent assay (ELISA) in the OP group. (D and E) Heatmap showing the expression of lipid sets associated with Cer and CE in OP tissues identified by lipid metabolomics. (F) Violin plot showing the expression levels of *CD36*, *CES1* and *SCD1* in the 13 clusters of OP. (G) Spatial expression of *CD36*, *CES1* and *SCD1* in the representative Visium CytAssist sample. The genes tended to be weakened in the OP area. (H) Immunohistochemical staining was used to analyse multiple molecules (CD36, CES1 and SCD1) in the lungs of OP patients. (I) UMAP of 4088 macrophages which were divided into three subclusters, including SCGB3A1^+^ epithelial phagocytic macrophages, VCAN^+^ monocyte‐derived macrophages (Mo‐AM, predominantly M1 subtype) and FABP4^+^ alveolar macrophages (resident macrophage TRAM, predominantly M2 subtype). (J) Dot plot showing the expression levels of marker genes for different subtypes of macrophages in the subclusters of macrophages. (K) Proportion of macrophage types in healthy control (HC) and OP samples. (L and M) Heatmap showing the expression of lipid sets associated with Cer (L) and CE (M) in FABP4^+^ lipid metabolising macrophage population identified by lipid metabolomics. (N) Lipid drops staining with Biotium/LipidSpot 488 Lipid Droplet Stain (1000×/70065‐T/20‐µL) in FABP4^+^ macrophages of the OP mouse model. (O) qRT‐PCR was used to detect the difference in expression levels of *CD36*, *CES1* and *SCD1* in lipid metabolising macrophage population of FABP4^+^. (P and Q) Western blot assay was used to detect the expression of CD36, CES1 and SCD1 in lipid metabolising macrophage population of FABP4^+^.

The above results revealed that lipid metabolism reprogramming, inflammatory response, immune system process and regulation of epithelial cell proliferation were involved in the conversion of monocytes into macrophages in OP patients. The lipid metabolomics analysis of lung tissue was performed to investigate the effect of OP on metabolomics, revealing that the metabolite levels were different between HC and OP groups (Figure 4A). Indeed, 98 lipid metabolites were significantly increased, while 68 lipid metabolites were decreased, as revealed by the volcano plot of the metabolomic analysis (Figure 4A, *p* < .05, FC > 2). Next, the different levels of these 166 lipid species were classified, demonstrating that the highest lipid species were cholesterol esters (CE, 25%) and Cer (18%) (Figure 4B). The total levels of CE and Cer were significantly increased in OP tissues (Figure 4C). The lipid species of CE and Cer were also detected and measured in OP, as shown in Figure 4D,E. C16, C18 and C20 Cer, which include a single or double bond were significantly decreased in OP, while C21, C22, C23 and C24 Cer were increased in OP (Figure 4D). Among them, the levels of most CE species were increased in OP tissues, such as CE (16:1), CE (17:0), CE (18:0) and CE (22:0) (Figure 4E). The previous proteomics, scRNA‐seq and spatial transcriptomics results revealed that the development of OP was closely related to lipid metabolism reprogramming and was mainly regulated by macrophages (Figures 2A‒D and 3). Furthermore, Figure 1H shows that lipid metabolism‐related genes, such as *CD36*, *CES1* and *SCD1*, were highly expressed in macrophages. Next, the expression of *CD36*, *CES1* and *SCD1* was measured in different cell types in HC and OP groups. Interestingly, CD36‐positive cells were significantly decreased in monocytes, macrophages and epithelial cells. *CES1* and *SCD1*‐positive cells were decreased in macrophages and epithelial cells (Figure 4F). Spatial transcriptomics also revealed that the expression of *CD36*, *CES1* and *SCD1* in the OP area was significantly lower than that in the surrounding normal tissues (Figure 4G). The immunohistochemistry results further proved that the intensity of *CD36*, *CES1* and *SCD1* in the OP area was significantly lower than that in the surrounding normal tissue (Figure 4H). Macrophages were classified into three subtypes (Figure 4I,J), that is SCGB3A1^+^ epithelial phagocytic macrophages, FABP4^+^ macrophages (resident macrophages, predominantly M2 subtype) and VCAN^+^ monocyte‐derived macrophages (predominantly M1 subtype). Among them, the number of FABP4^+^ and VCAN^+^ macrophages was decreased in the lung of OP patients, especially FABP4^+^ macrophages. An OP mouse model was used, alveolar macrophages were extracted (mainly resident macrophage/FABP4^+^ macrophages),^25^, ^26^ and lipid metabolomics analysis was performed (Figure 4K). FA and Cer (20‒24) levels were significantly decreased; Cer (16 and 18) (also called C16 and C18 Cer) levels were significantly increased. Most CE levels, especially CE 17:1/20:1/20:3/22:0/22:6, were higher in the alveolar macrophages of OP. In addition, CE (18:2) was significantly reduced in the alveolar macrophages of OP (Figure 4L). The fluorescence intensity of lipid droplets in alveolar macrophages of the mouse OP model was significantly decreased compared to that in the control mice, consistent with the findings from OP tissue staining (Figure 4M). Furthermore, a notable reduction in the expression of *CD36*, *CES1* (involved in the hydrolysis of CE) and *SCD1* (catalysing the synthesis of monounsaturated fatty acids [MUFA], mainly oleic acid [C18:1] and palmitoleic acid [C16:1]) was observed by RT‐qPCR and Western blot, which are molecules involved in the regulation of lipid metabolism in the alveolar macrophages of OP (Figure 4N‒Q). Based on the single‐cell sequencing analysis, it was observed that CD36, SCD and CES1 exhibited high expression levels in human lung FABP4^+^ macrophages, while their expression levels were nearly negligible in SCGB3A1^+^ macrophages (Figure S9A‒C). The prevalence of VCAN^+^ macrophages remained consistent between HC and OP groups (Figure 4K). These suggested that FABP4^+^ macrophages potentially modulate fatty acid uptake in individuals with OP through CD36, SCD and CES1 (Figures 4K and S9). In summary, CE might be present as lipid droplets in the FABP4^+^ macrophages of human lung to store and transport cholesterol and FA. Our results showed the decrease in the expression of CD36, leading to a reduced FA intake into FABP4^+^ macrophages. As regards CE, CE (18:2) level decreased, and the content of CE 17:1/20:1/20:3/22:0/22:6, which are regulated by CES1 increased in alveolar macrophages of OP. FA and C20–C24 Cer levels were significantly decreased, while C16 and C18 Cer levels were significantly increased.

### 3.4 Low level of CD36 promotes ferroptosis by attenuating the GSH/SLC3A2/GPX4/FTH1/FTL axis through lipid metabolism reprogramming in alveolar macrophages

**FIGURE 5:**
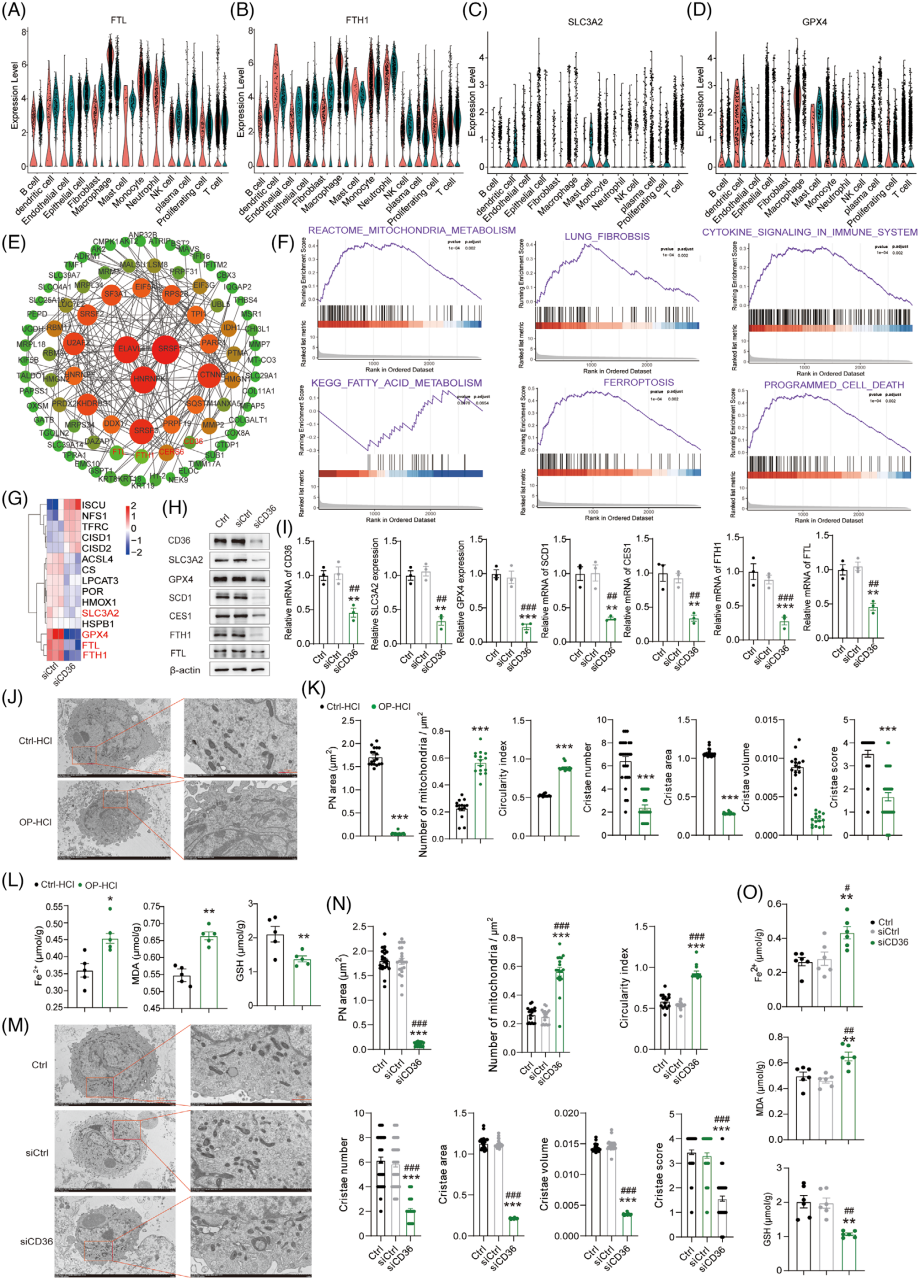
Reprogramming of lipid metabolism in alveolar macrophages promotes ferroptosis. (A‒D) Violin plot showing the expression levels of *FTL* (B) and *FTH1* (C) in the 13 clusters of healthy control (HC) and organising pneumonia (OP) tissues from the single‐cell RNA sequencing (scRNA‐seq) data. (E) Network of differentially expressed proteins between CON and siCD36 groups (red represents upregulation and green represents downregulation). (F) Gene set enrichment analysis (GSEA) analysis revealed that the changed proteins were mainly enriched in lung fibrosis, mitochondria‐related metabolism, fatty acid metabolism, immune system, cell death and ferroptosis‐related pathways by knocking down CD36. (G) Heatmap showing the differentially expressed proteins related to ferroptosis in alveolar macrophages by knocking down CD36. (H and I) Western blot assay was used to detect the expression of CD36, SLC3A2, GPX4, FTH1, FTL, CES1 and SCD1 in alveolar macrophages. (J) Electron micrograph of alveolar macrophages of OP mouse model (magnification, 30 000×; red arrow, mitochondrion; red rectangle, nucleus). (K) Quantification of perinuclear mitochondrial area, number of mitochondria per square micron, mitochondrial circularity index, cristae number, cristae area, cristae volume and cristae score from (J). (L) The concentration of Fe^2+^, malondialdehyde (MDA) and glutathione (GSH) in alveolar macrophages of OP mouse model. (M and N) Electron micrograph of alveolar macrophages with siCD36 treatment. Quantification of perinuclear mitochondrial area, number of mitochondria per square micron, mitochondrial circularity index, cristae number, cristae area, cristae volume and cristae score from (M). (O) The levels of Fe^2+^, MDA and GSH in alveolar macrophages with siCD36 treatment. Statistical significance is indicated by asterisks; ^***^ *p* ≤ .001.

The above results demonstrated that the reprogramming of lipid metabolism in OP tissue and the reduction of the number of macrophages were related to ferroptosis. Our hypothesis was that the above decrease in the number of macrophages might be due to the regulation of genes related to ferroptosis through the reprogramming of lipid metabolism, thus promoting the ferroptosis of macrophages. The proteomic analysis of the OP tissue indicated a significant increase in the expressions of ferritin light chain (*FTL*), ferritin heavy chain 1 (*FTH1*), *SLC3A2*, *GPX4*, *ACO1* and *ISCU*, potentially associated with its abnormal proliferation (Figure S10A). However, scRNA‐seq demonstrated a significant decrease in *FTL*, *FTH1*, *SLC3A2* and *GPX4* expression specifically in OP macrophages, while their expression was significantly increased in neutrophils (Figure 5A‒D). In the OP tissue, there was an overall increase in the levels of FTL and FTH1 (Figure S10A), possibly attributed to the existence of an abnormally proliferating plasma cell population. Nonetheless, Figure 5A,B illustrates a notable reduction in FTL, FTH1, SLC3A2 and GPX4 levels in macrophages. Lipid metabolism reprogramming has the potential to decrease the expression of ferritin *FTL* and *FTH1*, consequently increasing the intracellular iron content and inducing ferroptosis.^27^, ^28^ Therefore, our hypothesis was that the downregulation of SLC3A2, GPX4 and FTH1/FTL (ferritin component) in alveolar macrophages of OP facilitated ferroptosis.

The effect of CD36 knockout was evaluated in alveolar macrophages (FABP4^+^ macrophages). The hierarchical clustering analysis of proteins with differential expression revealed significant changes in the expression of 128 proteins (Figure S10B). Additionally, the protein read count expression profile was subjected to further analysis using volcano plots, which identified 62 upregulated proteins and 66 downregulated proteins in the siCD36 group compared to the siCtrl group (Figure S10C). Next, GO‒KEGG analyses were performed to understand the significance of the difference in protein expression between siCD36 and siCtrl group. Our findings indicated that the proteins that changed in the siCD36 group were involved in pathways related to regulation of RNA splicing, cellular response to oxidative stress, lipid transport, lipid localisation, cell death and immune process, which is probably regulated by membrane bounded organelle, mitochondrion and ferritin complex (Figure S10D). Furthermore, the KEGG analysis revealed that the signalling pathways primarily associated with ferroptosis were more prevalent in the siCD36 group compared to the siCtrl group (Figure S10D).

CD36 regulated the expression of CERS6, FTL and FTH (Figure 5E), as revealed by the analysis of gene network signal map. Next, GSEA on the changed proteins showed that secretome of lung fibrosis, mitochondria metabolism, cytokine signalling in immune system, cell death and ferroptosis‐related pathways were activated by *CD36* knockout (Figure 5F). GPX4 converts glutathione (GSH) to oxidised GSH and reduces the cytotoxic lipid peroxide to the corresponding alcohol. The inhibition of GPX4 expression in alveolar macrophages with CD36 knockdown led to the accumulation of lipid peroxides, which is a marker of ferroptosis (Figure 5G). The downregulation of SLC3A2 reduced the content of cysteine and GSH, inducing the accumulation of ROS and the occurrence of ferroptosis in alveolar macrophages with CD36 knockdown (Figure 5G). Additionally, our investigation revealed a noteworthy reduction in the expression of SLC3A2, GPX4, FTL and FTH1 associated with ferroptosis after the interference with CD36 expression in alveolar macrophages (Figure 5H,I). Meanwhile, in RAW264.7 cell lines, the knockdown of CD36 led to reduced expression of SLC3A2, GPX4, FTL and FTH1, all of which are known to be associated with ferroptosis (Figure S10E,F). Electron microscopy revealed ultra‐morphological features indicating cell membrane rupture and vesiculation, mitochondrial reduction, increased membrane density, reduced or absent mitochondrial ridges, rupture of the mitochondrial outer membrane and normal nuclear size with the absence of chromatin condensation in alveolar macrophages of the OP mice and siCD36 macrophages. In the OP mouse model, a remarkable reduction was observed in the mean mitochondrial surface area surrounding the nucleus, implying mitochondrial clustering. Concurrently, a rise in mitochondrial density per micrometer squared and an enhancement in the typical roundness metric were documented, suggesting possible structural reorganisation. These observations indicate a reduction in mitochondrial fusion and an increase in fission events in the OP group. Disruption of cristae morphology was also observed, along with reductions in the average number of cristae per mitochondrion, average cristae surface area and cristae volume density in the OP group. Additionally, the cristae score was lower in the OP group compared to the HC group. These findings confirm the importance of the OP group in cristae remodelling and its disruption of the norm (Figure 5J,K). A series of ferroptosis indicators were assessed in alveolar macrophages to investigate our hypothesis. Silencing of CD36 led to increased levels of ferrous ions, elevated malondialdehyde (MDA), and reduced GSH in the alveolar macrophages of OP mice (Figure 5L). Specifically, we demonstrated that knockdown of CD36 resulted in a significant decrease in the mean mitochondrial area of the perinuclear mitochondria cluster. Furthermore, the absence of CD36 was found to significantly alter mitochondrial dynamics, as evidenced by an augmented density of mitochondria per micrometer squared and a shift towards more circular shapes. This implies impaired fusion processes and a stimulation of fission. Morphological disturbances in cristae structure were confirmed, accompanied by a decrease in the mean cristae count per mitochondrion, cristae surface area and cristae volume fraction. The diminished cristae score in CD36‐deficient cells further accentuates this disruption relative to controls, underscoring the pivotal function of CD36 in cristae architecture and its indispensable role in maintaining cellular equilibrium (Figure 5M,N). A series of ferroptosis indicators were measured in alveolar macrophages to test our speculation. The silencing of CD36 increased ferrous ions, increased MDA and decreased GSH in the alveolar macrophages of OP mouse and siCD36 macrophages (Figure 5O). These results suggested that CD36 knockdown promoted ferroptosis by regulating lipid metabolism, specifically by reducing the expression of SLC3A2, GPX4, FTH1 and FTL and mainly through the classical GSH/SLC3A2/GPX4/FTH1/FTL pathway.

Ferroptosis is an active cell death, and since ribosomes were analysed in omics, proteins relevant to ferroptosis were selected for re‐analysis. Proteins related to energy metabolism and ribosome significantly increased in the OP group (Figure S11A‒C). In addition, the intracellular ATP in alveolar macrophages was increased in the OP mouse model (Figure S11D), as revealed by the CellTiter‐Glo Cell Viability Assay. According to previous report, CD36 regulates the lipid metabolism of fatty acid.^29^ The measurement of proteins related to energy metabolism (proteins related to energy metabolism including ATP and mitochondrion) and ribosomes revealed that the expression of many of them was significantly decreased in the siCD36 group, while the expression of other proteins (Figure S11A‒C) showed no significant difference (Figure S11E). In addition, the level of intracellular ATP was increased in the siCD36 group (Figure S11F). Mitochondria accumulate ROS on their membrane through proton electrochemical gradient potential to promote ferroptosis and produce ATP. The interference with CD36 promoted ferroptosis in macrophages, while the ATP content increased. In summary, the upregulation of C16 Cer synthesis in OP aggravated CD36^−/−^‐induced mitochondrial dysfunction and ferroptosis in macrophages. Thus, the protection of mitochondria might attenuate ferroptosis in macrophages by reducing Cer content in OP.

### 3.5 High level of C16 Cer promotes ferroptosis by attenuating the FTH1/FTL axis through lipid metabolism reprogramming in alveolar macrophages

**FIGURE 6:**
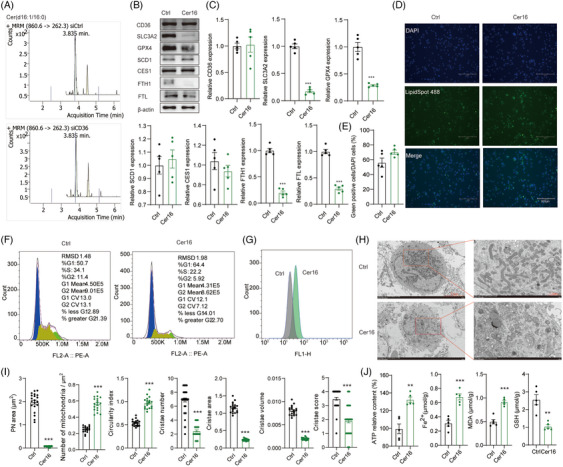
The impact of C16 ceramide (Cer) on ferroptosis in alveolar macrophages. (A) The content of C16 Cer with CD36 interference in alveolar macrophages in mass spectrometry analysis. (B) Western blot assay was used to detect the expression of CD36, CES1 and SCD1 in alveolar macrophages with C16 Cer treatment. (C) Perform statistical analysis on the Western blot of (B). (D and E) Lipid drops staining with Biotium/LipidSpot 488 Lipid Droplet Stain (1000×/70065‐T/20‐µL) in alveolar macrophages with C16 Cer treatment. (F) Perform cell cycle analysis on alveolar macrophages treated with C16 Cer. (G) Intracellular reactive oxygen species (ROS) measurement by flow cytometry after two compounds treatment (Marker, BODIPY 581/591 C11). (H and I) Electron micrograph of alveolar macrophages with C16 Cer treatment. (J) The levels of Fe^2+^, malondialdehyde (MDA) and glutathione (GSH) in alveolar macrophages with C16 Cer treatment.

The silencing of CD36 resulted in an increased presence of C16 Cer in alveolar macrophages (Figures 6A and S12), as revealed by mass spectrometry. The function of C16 Cer was corroborated by discovering that the expression of CD36, SCD1 and CES1 remained relatively unaltered after the incubation with C16 Cer. However, a significant reduction in the expression of SLC3A2, GPX4, FTH1 and FTL was observed (Figure 6B,C). Concurrently, alveolar macrophages incubated with C16 Cer (Cer16) displayed lipid droplet staining, with a marginal increase in their count, although without any significance (Figure 6D,E). Additionally, a substantial inhibition was observed in the proliferation of alveolar macrophages after the incubation with C16 Cer (Figure 6F). A comprehensive evaluation of ferroptosis indicators was carried out in alveolar macrophages to confirm our hypothesis. C16 Cer enhanced the intracellular ATP content and ferrous ion content, facilitated lipid peroxidation, increased MDA levels and decreased GSH levels (Figure 6G). In this study, we have shown that elevated levels of Cer16 lead to a notable reduction in the average mitochondrial area within the perinuclear mitochondria cluster. Additionally, an increase was noted in both the quantity of mitochondria per square micron and the mean circularity index. These results indicate that heightened Cer16 levels impede mitochondrial fusion while encouraging fission events. Our analysis also confirmed disturbances in cristae morphology, evidenced by decreases in the average number of cristae per mitochondrion, average cristae surface area, and cristae volume density in the Cer16 group. Furthermore, the cristae score was lower in the Cer16 group compared to the HC group. These findings underscore the essential role of Cer16 in cristae restructuring and the disruptive effects of low CD36 expression on cellular homeostasis (Figure 6H,I). Moreover, the ultrastructural morphology was indicative of cell membrane rupture and vesiculation, mitochondrial reduction, increased membrane density, decreased or absent mitochondrial ridges, rupture of the mitochondrial outer membrane and normal nuclear size without chromatin condensation in alveolar macrophages with increased levels of C16 Cer (Figure 6G‒J).

### 3.6 Alveolar macrophage secretions contribute to epithelial fibrosis in OP

**FIGURE 7:**
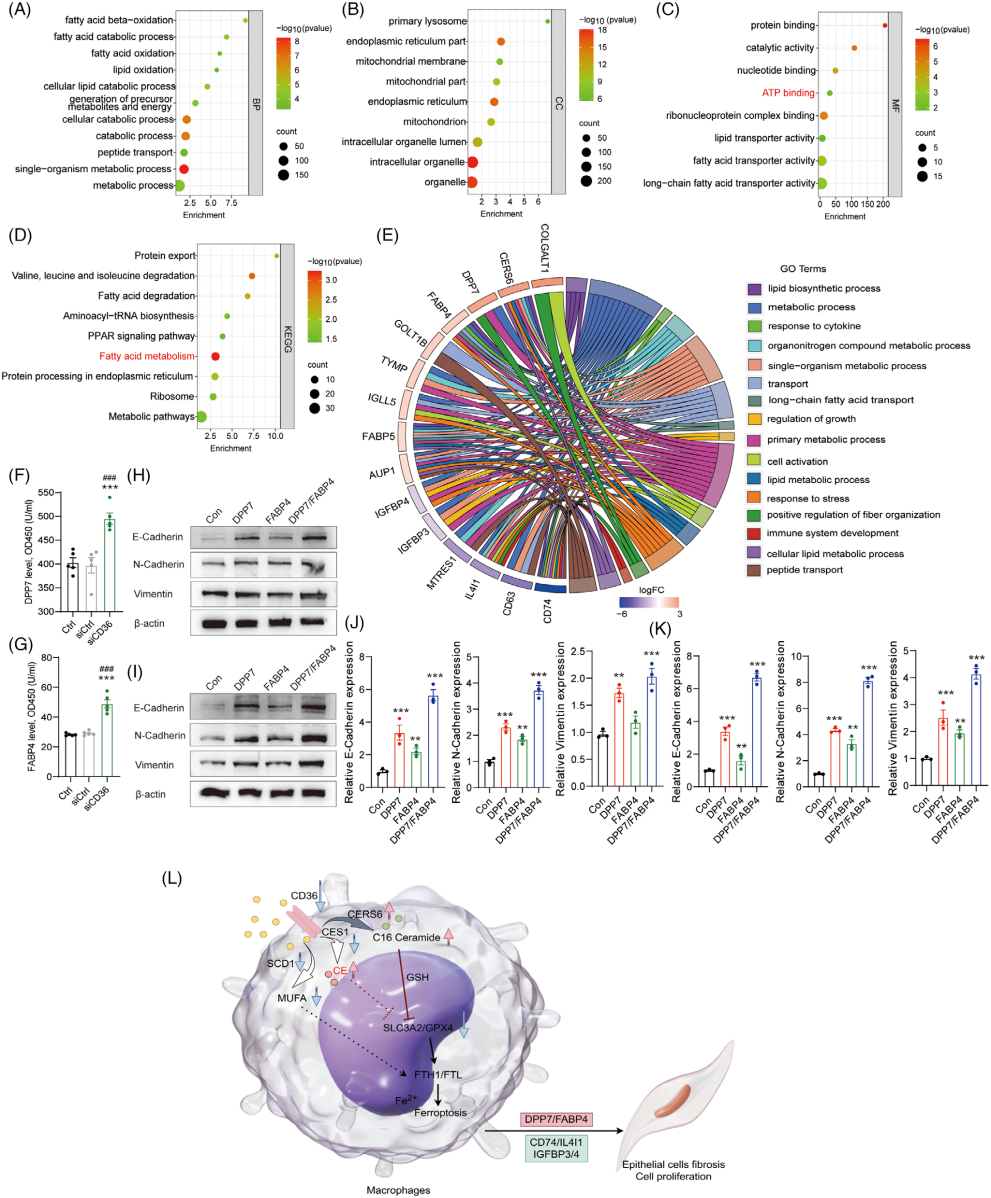
Alveolar macrophage secretions promote epithelial fibrosis. (A‒D) Gene Ontology (GO) enrichment analysis and Kyoto Encyclopedia of Genes and Genomes (KEGG) analysis for the categories of upregulated and downregulated secretions between CON and siCD36 groups. (E) The chord of GO showed the dramatically differential secretions, which regulated epithelial fibrosis and proliferation. (F and G) The levels of DPP7 and FABP4 in alveolar macrophages with siCD36 treatment. (H‒K) Western blot assay was used to detect the expression of E‐cadherin, N‐cadherin and vimentin in alveolar macrophages (H and J: MLE‐12; I and K: Beas2B cell lines) with DPP7 or FABP4 treatment. (L) A schematic of ferroptosis in alveolar macrophages and alveolar macrophage cell secretions contributing to epithelial fibrosis of organising pneumonia (OP) via CD36‐related pathway.

The secretion of alveolar macrophages was measured to investigate the impact of alveolar macrophages on epithelial fibrosis and found significant alterations in the expression of 184 proteins by the hierarchical clustering analysis (Figure S13A, FC > 2, *p *< .05). The protein read count profile was further analysed using volcano plots, revealing 86 upregulated proteins and 98 downregulated proteins in the OP compared to the HC group (Figure S13B). The upregulation of CD74 was associated with the regulation of most secreted proteins (Figure S13C), as revealed by gene network signal map. GO‒KEGG enrichment analysis showed that alveolar macrophage secretion was significantly increase in pathways related to metabolic process, including fatty acid catabolic process by regulating mitochondrion and organelle through ATP binding, lipid transporter activity and long‐chain fatty acid transporter activity (like C24 Cer) (Figure 7A‒D). The analysis of the supernatant of alveolar macrophages after CD36 knockdown was consistent with its cell precipitation data, further confirming that CD36 inhibited the ferroptosis *SLC3A2/GPX4* pathway in alveolar macrophages in OP tissue by promoting lipid metabolism (CE, C16/C24 Cer). Additionally, the significantly regulated secretion was analysed using GO. The significantly upregulated COLGALT1, dipeptidyl peptidase (DPP7) and *FABP4/5* exerted a positive regulation of fibre organisation and epithelial cell proliferation, while the downregulation of interleukin‐4‐induced‐1 (IL4I1) also promoted fibre organisation (Figure 7E). COLGALT1 promotes the galactose modification of COL3A1 and further aggravates the fibrotic cascade. DPP7 inhibitors ameliorate kidney fibrosis,^30^ suggesting that high DPP7 expression promotes kidney fibrosis. The fatty acid‐binding protein 4 (FABP4) contributes to renal interstitial fibrosis by mediating inflammation and lipid metabolism,^31^ thus exacerbating cardiac fibrosis.^32^ Immunostimulatory cytokines, including IL4I1, are downregulated in macrophages with decreased CD74 expression. The oncogenic effect of IL4I1 is likely mediated by local anti‐ferroptotic pathways through aromatic amino acid metabolism.^33^ The downregulation of *IL4I1* also promotes ferroptotic pathways in macrophages of OP. Xiao et al. reported that blocking *CD74* in macrophages weakens the anti‐tumour activity and proliferation ability of CD8 CTLs in hepatocellular carcinoma.^34^ Increased thymidine phosphorylase (TYMP) in siCD36 macrophages is related to metabolic processes promoting liver fibrosis.^35^, ^36^ IGFBP3/4 are downregulated by siCD36. The upregulation of AUP1 and downregulation of IGFBP3 expression in liver cells are associated with the development of liver fibrosis and cirrhosis.^37^, ^38^ Macrophages promote cardiac fibrosis by upregulating cytokines and growth factors associated with the development of cardiac fibrosis.^39^, ^40^ CERS6 promotes C16 Cer synthesis and is upregulated by the inhibition of CD36 in macrophages. GOLT1B promotes the peptide transport in macrophages of OP, which is also correlated with a worse prognosis in lung adenocarcinoma.^41^ IGLL5 is correlated with the pathogenic mechanism and prognosis of multiple diseases.^42^, ^43^ CD63 is also related to myocardial fibrosis.^44^ CERS6, FABP4, IGLL5, AUP1, IGFBP3/4, IL4I1 and CD74 are related to the regulation of macrophage growth in the CD36 deficiency group, and all these proteins are related to metabolic processes,^41^, ^42^, ^43^, ^44^ including lipid biosynthetic process, primary metabolic process, lipid metabolic process, long‐chain fatty acid transport and cellular lipid metabolic process. Furthermore, a significant increase in the secretion levels of DPP7 and FABP4 confirmed using ELISA was observed following the knockdown of CD36 (Figure 7F,G). This observation aligns with the results of the proteomic analysis conducted. Subsequently, normal human bronchial epithelial cells (Beas2B) and mouse pulmonary epithelial cells (MLE‐12) were stimulated with secretory factors DPP7 and FABP4, resulting in a notable upregulation in the expression levels of fibrosis‐associated proteins E‐cadherin, N‐cadherin and vimentin (Figure 7H‒K). Therefore, our hypothesis is that the sustained lack of CD36 in OP macrophages affects the ferroptosis pathway GSH/SLC3A2/GPX4/FTH1/FTL, promoting the secretion of DPP7 and FABP4, and thus facilitating fibrosis of epithelial cells (C16 Cer) (Figure 7I).

## 4 DISCUSSION

Corticosteroid therapy is commonly recommended for the management of OP, but the increasing prevalence of the condition necessitates exploring alternative treatments. This study describes a comprehensive approach integrating proteomics, mass spectrometry, scRNA‐seq and Visium CytAssist methodologies to elucidate the intricate immunological landscape of OP at a high resolution. This investigation offers a potential new perspectives in the management of OP and its therapeutic strategies.

Our findings revealed the substantial presence of different immune cell populations within the OP tissue. A significant increase in the proportion of B cells and plasma cells was observed, while the proportions of macrophages, NK cells and T cells were decreased. Furthermore, DEGs associated with lipid metabolism were identified in these immune cells, specifically regulating lipid localisation, transport and cholesterol synthesis. Subsequently, the role of macrophages in OP tissue was investigated, revealing the most pronounced changes in the subpopulation of FABP4^+^ macrophages (alveolar macrophages). The expression of CD36 in FABP4^+^ macrophages caused various abnormalities in lipid metabolic reprogramming, such as increased levels of C16 Cer and CE. Additionally, CD36 expression facilitated ferroptosis through the SLC3A2/GPX4 pathway and its regulation of COLGALT1, DPP4 and FABP4/5 promoted the fibrosis of epithelial cells and the dysplasia of OP tissue. A reduction in the expression of CD36 was found in OP patients, subsequently leading to a decline in SCD1 expression. CD36 plays a crucial role in regulating lipid balance by preferentially absorbing MUFA during extracellular matrix detachment and tumour advancement.^45^ This sequence of events contributed to a decrease in MUFA levels, which effectively stimulated non‐apoptotic ferroptosis and iron‐dependent oxidative cell death. A decrease in CD36 levels also resulted in a reduction in CES1 expression, thereby causing an increase in CE levels. Silencing CES1 expression in human THP‐1 macrophages was found to reduce cholesterol uptake and decrease CD36 expression in cells loaded with acetylated low‐density lipoprotein.^46^ This inhibited the expression of SLC3A2 and GPX4, hence promoting ferroptosis. Additionally, the low CD36 expression facilitated the increase in CERS6 expression, which is involved in the synthesis of C16 Cer. This subsequently resulted in the reduction of GSH levels, thereby promoting the ferroptosis pathway. These three pathways collectively stimulated non‐apoptotic ferroptosis and iron‐dependent oxidative cell death in alveolar macrophages of OP patients. The cell death in alveolar macrophages induced the increase in DDP7 and FABP4 expression, while causing a decrease in CD74, IL4I1 and IGFBP3/4 expression. These alterations in released factors contribute to fibrosis and cell proliferation in surrounding epithelial cells of OP. Fibrosis represents the ultimate outcome of virtually all types of chronic diseases, regardless of their underlying causes.^47^ The efficacy of a vaccination inducing CD8 T cell to prevent or treat pulmonary fibrosis, has been proved as effectively mitigating the presence of fibroblasts and subsequent fibrosis in the pulmonary region of mice.^48^ These findings establish a potential use of vaccination‐based immunotherapies to combat fibrosis.^49^ Our results suggested that the macrophage secretion of COLGALT1 might influence epithelial cell fibrosis. The massive secretion of COLGALT1 in macrophages promoted the galactose modification of COL3A1, thus further aggravating the pulmonary fibrosis cascade. The downregulation of CD74 and of multiple immunostimulatory cytokines increases the rate of liver fibrosis and the proliferation of liver tumours.^34^, ^50^ The low expression of CD74 in CD36‐knockdown macrophages led to the downregulation of multiple secreted immunostimulatory cytokines (IL4I1, CD63 and IGFBP3/4), which was potentially related to fibrosis and proliferation of OP tissue. Our results suggest that macrophages may influence the fibrosis of lung epithelial cells through the secretion of DPP7 and FABP4, although further intervention studies targeting DPP7 and FABP4 are needed to confirm this (Figure 7).

The maintenance of cholesterol homeostasis is crucial for the overall functioning of the body, involving a delicate balance between cholesterol synthesis, uptake, transport, storage and metabolism. Sterol O‐acyltransferase 1 (SOAT1) in cells facilitates the conversion of newly synthesised cholesterol into CE, while acyl coenzyme A cholesterol acyltransferase 1 (ACAT1) is responsible for converting the excess of free cholesterol into CE and storing it in lipid droplets.^51^, ^52^ A notable increase in the expression of ACAT1 was observed in non‐small cell lung cancer.^53^ This finding suggested that an excess of ACAT1 in these cells facilitated/facilitates the conversion of cholesterol into CE, leading to an abnormal increase in the concentration of CE within the body and a consequent breakdown of the steady‐state equilibrium.^54^ Subsequent in vitro cellular investigations demonstrated that the disruption of ACAT1 expression effectively inhibited the proliferation and invasion of Lewis lung cancer cells, thereby retarding the growth of cancer cells. The aforementioned experiments demonstrated that CE, rather than cholesterol, represents the promoting agent of tumour proliferation. Several studies revealed a substantial reduction in CYP27A1 expression, which is associated with CE metabolism, in lung adenocarcinoma cells compared to healthy cells. Moreover, a negative correlation exists between CYP27A1 expression and the extent of metastasis of lung adenocarcinoma cells.^55^

In addition to its influence on survival of lung cells, CE anabolism is also associated with Cer expression, which is involved in lung diseases. Among the various Cer found in lung cells, palmitoyl Cer (C16 Cer, Cer16) and lignin polyarylceramide (C24 Cer) are the most prevalent; the former induces cell apoptosis, and the latter protects against cell apoptosis. High C16 Cer is associated with pulmonary vascular cell death and subsequent pulmonary vascular impairment.^56^ Furthermore, a positive correlation exists between the expression of C16 Cer and the severity of the COVID‐19 disease.^57^ Silencing Cer synthase 6 (CERS6) inhibits the migration of A549 cells, which is reversed by the addition of exogenous C16 Cer, suggesting the increase in the migratory ability of A549 cells compared to that of the same cells lacking C16 Cer, thus indicating that C16 Cer increases the migration of cancer cells.^58^, ^59^ Hammerschmidt et al. found that the synthesis of sphingolipids dependent on CERS6 in hypothalamic neurons leads to endoplasmic reticulum/mitochondrial stress, resulting in impaired glucose homeostasis in obese mice.^60^ As regards Cer C18, its levels are higher in obese asthma patients compared to non‐obese asthma patients, thereby indicating a potential association between obesity and asthma susceptibility. Therefore, Cer C18 may serve as a catalyst in the development of obesity‐induced asthma.^61^ In conclusion, the maintenance of Cer stability is crucial in the maintenance of a good lung health, and any disturbances in its metabolism increase the risk of developing respiratory disorders. C16:0 Cer induces apoptosis in Atgl^‒/‒^ macrophages by activating the mitochondrial apoptotic pathway.^62^ Our study revealed that CERS6 upregulation significantly enhanced the production of C16 Cer in the siCD36 group, which showed a higher ratio of C16 Cer to C24 Cer.

Ferroptosis is a novel form of cell death that was defined recently. Oxidative damage to cell membranes is initiated by the accumulation of intracellular iron and lipid peroxidation.^63^ The circulating iron is transported into cells through the transferrin receptor 1, followed by conversion to ferrous iron and subsequent release into the cytoplasm by the divalent metal transporter 1.^64^ Our results showed an increase in intracellular Fe^2+^ in alveolar macrophages after CD36 silencing. Within the tumour microenvironment, CD36's facilitation of fatty acid uptake by CD8 T cells triggers exacerbated lipid peroxidation and ferroptotic events, compromising the release of cytotoxic cytokines and dampening their tumour‐suppressive capacity. Notably, the targeted suppression of CD36 expression rejuvenates the CD8 T cells’ anti‐tumour efficacy, thereby constraining tumour growth, highlighting a potential therapeutic avenue.^65^ CD36 expression in OP macrophages decreased, causing the death of many macrophages. Our hypothesis was that this death might be related to the ferroptosis of macrophages. GSH‒GPX4 is the classic pathway of ferroptosis metabolism, and paclitaxel downregulates SLCSA2 and GPX4, promoting ferroptosis.^66^ Our study demonstrated that the downregulation of CD36 markedly decreased CES1 and SCD1 in macrophages of OP, suggesting that CD36 might promote ferroptosis through the regulation of lipid metabolism. ROS are mainly produced in mitochondria, which participate in the regulation of oxidative balance, and undergo size decrease and outer membrane damage during ferroptosis.^67^ The synthesis of the selenoprotein GPX4 is associated with large subunit of ribosomes, which is often upregulated in proliferating cells.^68^ The downregulation of CD36 inhibited the expression of RPL31 and MRPL28, the components of the 60S ribosome and 39S mitoribosome, suggesting a potential impact on ribosome and mitochondrion functions. Consequently, the increased ratio of C16 Cer to C24 Cer resulted in the downregulation of the SLC3A2/GPX4 pathway and reduced expression of FTH1/FTL, consequently inducing iron‐induced cell death in alveolar macrophages.

## AUTHOR CONTRIBUTIONS

Ying Tang, Cuilin Chu and Hongmei Wang designed the research. Ying Tang, Sen Qiao and Hongmei Wang wrote the manuscript. Lingyan Huang provided relevant tissue samples of human OP for proteomics, metabolomics, scRNA sequencing, spatial transcriptomics and immunohistochemical testing. Ying Tang, Jianfeng Xie and Hongmei Wang analysed data related to scRNA sequencing, spatial transcriptomics, proteomics and metabolomics. Siyuan Bu, Qin Sun and Airan Liu contributed to research materials and some experiments related to cells. Ying Tang, Cuilin Chu and Hongmei Wang analysed results and data.

## CONFLICT OF INTEREST STATEMENT

The authors claim that it has not been and will not be submitted simultaneously to another journal, in whole or in part. If the paper is accepted, all the authors will observe the License to Publish terms and seek the consent of the publishers acting for the owners of the journal in any other circumstance. The authors declare no competing interest.

## ETHICS STATEMENT

The research on designing human tissues was approved by the Medical Research Ethics Review Committee of the General Hospital of Ningxia Medical University with the approval number KYLL‐2022‐66. Patients/subjects have signed informed consent forms. All experimental procedures were approved and performed in accordance with the ethical regulations and the animal welfare committees of Southeast University (20230801002). All efforts were made to minimise animal suffering and reduce the number of animals used.

## Supporting information

Supporting Information

## Data Availability

scRNA‐seq and spatial transcriptomics raw data and filtered expression matrix can be accessed in GEO under the accession numbers GSE250404 and GSE250385. The datasets used and/or analysed during the current study are available from the corresponding author on reasonable request.
