# Supplementary material for: Integrated multi‐omics profiling landscape of organising pneumonia
Source: Clin Transl Med. 2024 Jul 31;14(8):e1782. doi: 10.1002/ctm2.1782 (PMC11290555; doi:10.1002/ctm2.1782)
Supplement: Supplementary file 1 — Supporting Information [file CTM2-14-e1782-s001.docx]

Figure S1


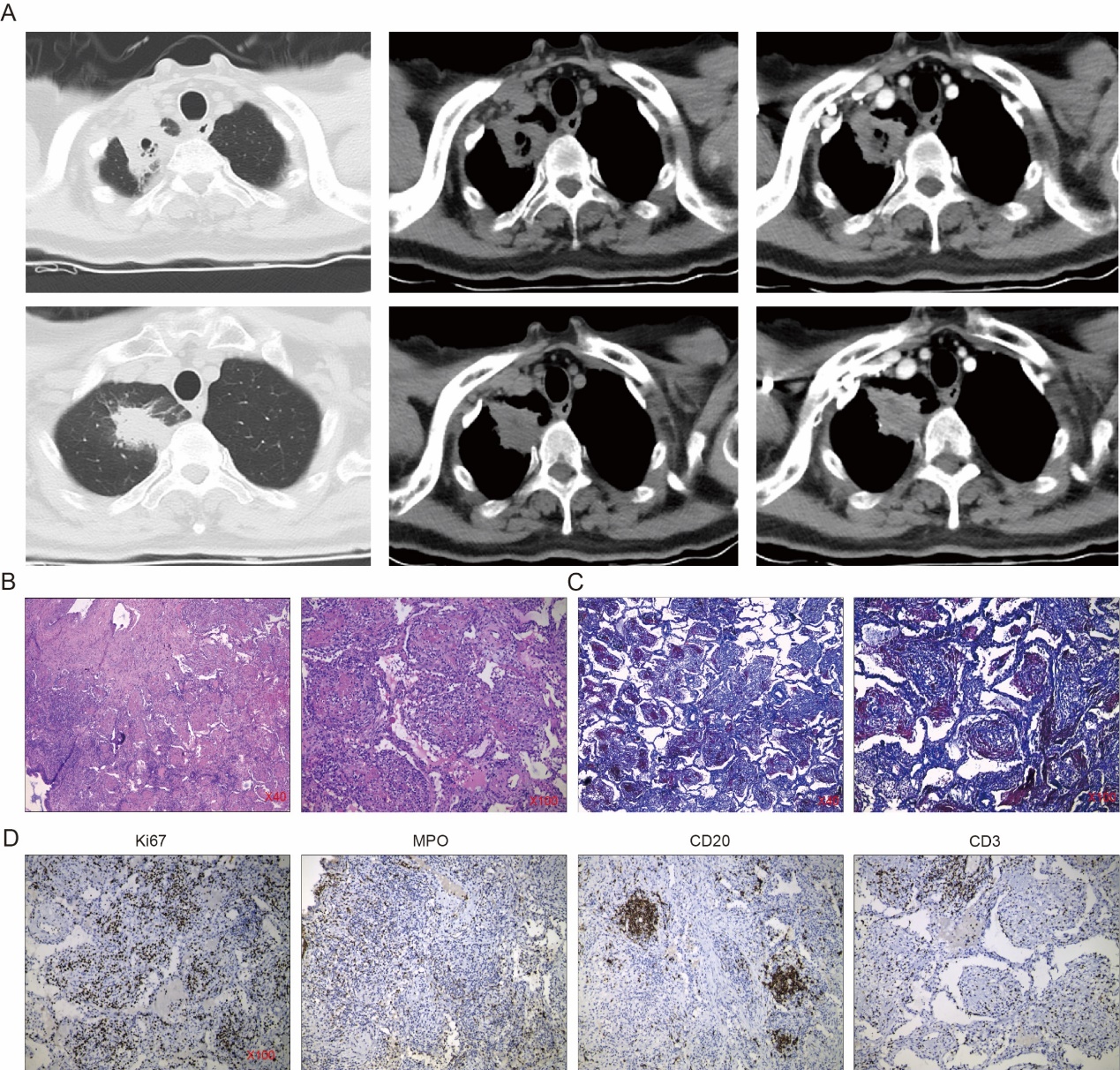


Figure S1. OP patient identification. (A) There was an irregular mass in the upper lobe tip segment of the right lung, with unclear boundary and surrounding burr signs. The cavity (blue arrow) and necrosis (red arrow) could be seen, and the lesion was significantly enhanced by enhanced scanning. (B) Fibroblastic thrombus nodules (red cycle) were seen in the lung surrounding the bronchus (green rectangle) and arteriovenous (blue rectangle), indicating organic pneumonia. A large number of lymphocytes were seen, with more abundant lymphocytes near the trachea and arteriovenous lung. These showed the fibroblast thrombus formation process in OP tissue. (C) Polypoid masses filled with granulation tissue in alveolar ducts and alveolar bronchioles (Masson bodies). (D) Ki67: Associated with cell proliferation. The more mature the OP plug, the higher the Ki67 in the plug. During the maturation process of OP, the more mucus, the more macrophages, the gradual death of alveolar cells, and the increase of suppository proliferation. MPO: Granulocyte +, scattered distribution, the airway lumen can be seen mucus pus thrombin. CD20: B lymphocytes +, most of them clustered and distributed, very few scattered distributions. CD3: T cells +, more than CD20+ B cells, aggregated and dispersed.

Figure S2


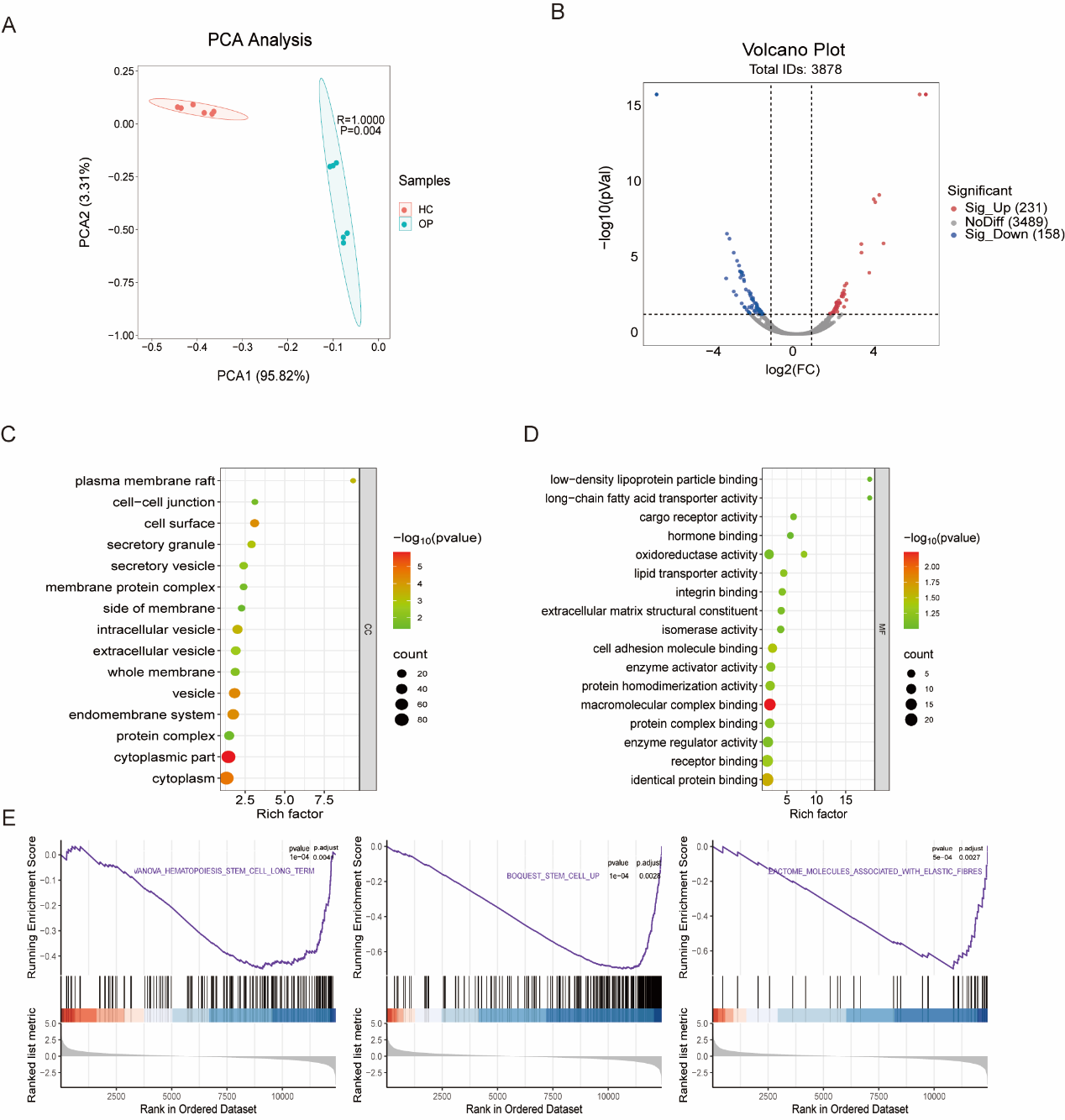


Figure S2. Quantitative proteomic analysis reveals distinct protein signatures in different groups. (A) PCA plot of proteomic data with two groups and 6 biological replicates each. (B) Volcano plots compare two groups as indicated in the plot. Groups with fold change >1.5 and p-value <0.05 were considered significantly differential expressions. The scatter plot depicts the difference score of these terms versus the −log10-transformed p-value, which is shown with red (UP) and blue (DOWN). (C-D) GO enrichment analysis (CC and MF) for the categories of upregulated and downregulated proteins between HC and OP groups. (H) GSEA analysis revealed that the proteins were mainly enriched in stem cell long term, stem cell up, and molecules associated with elastic fibers.

Figure S3


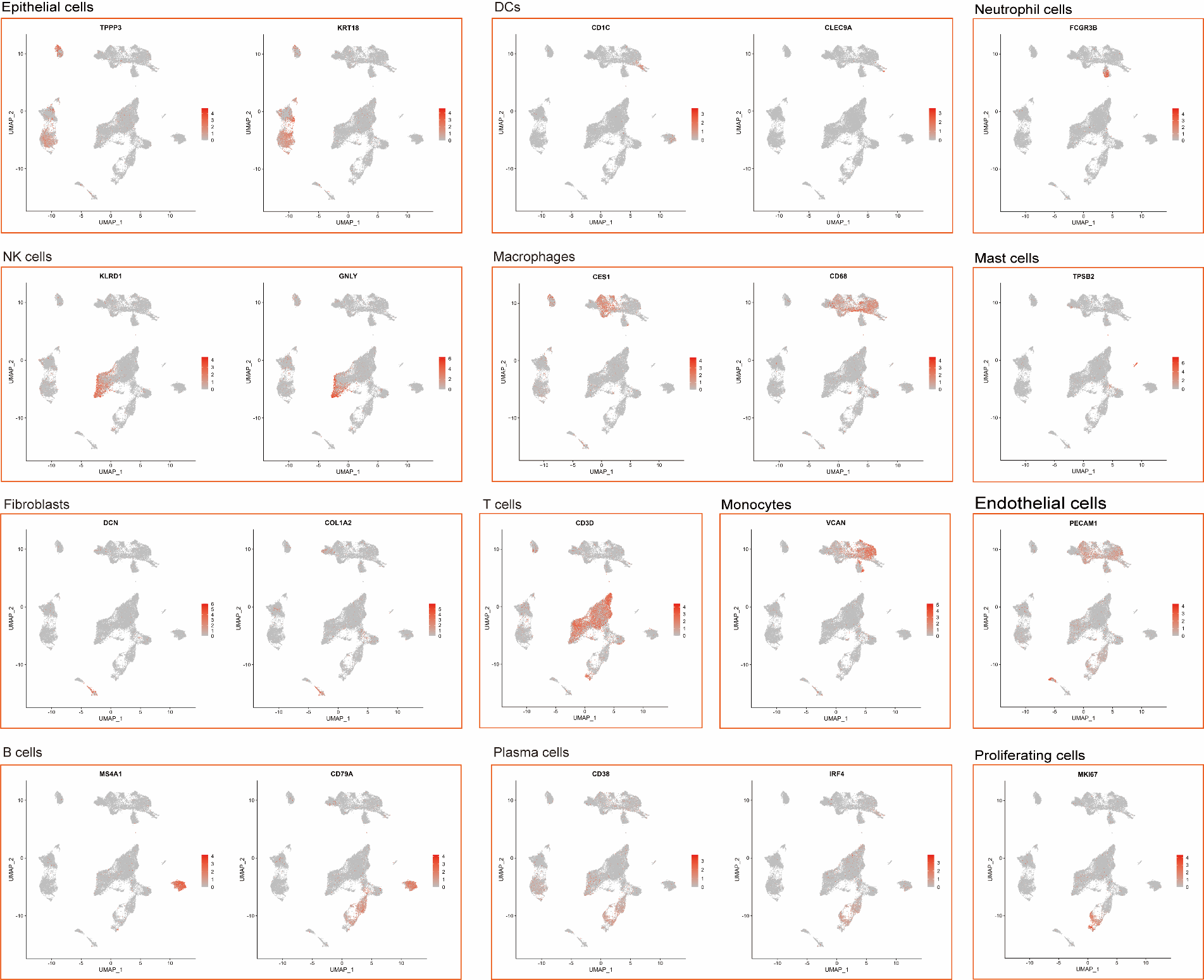


Figure S3. Using classical markers to cluster different cells in OP tissue.

Figure S4


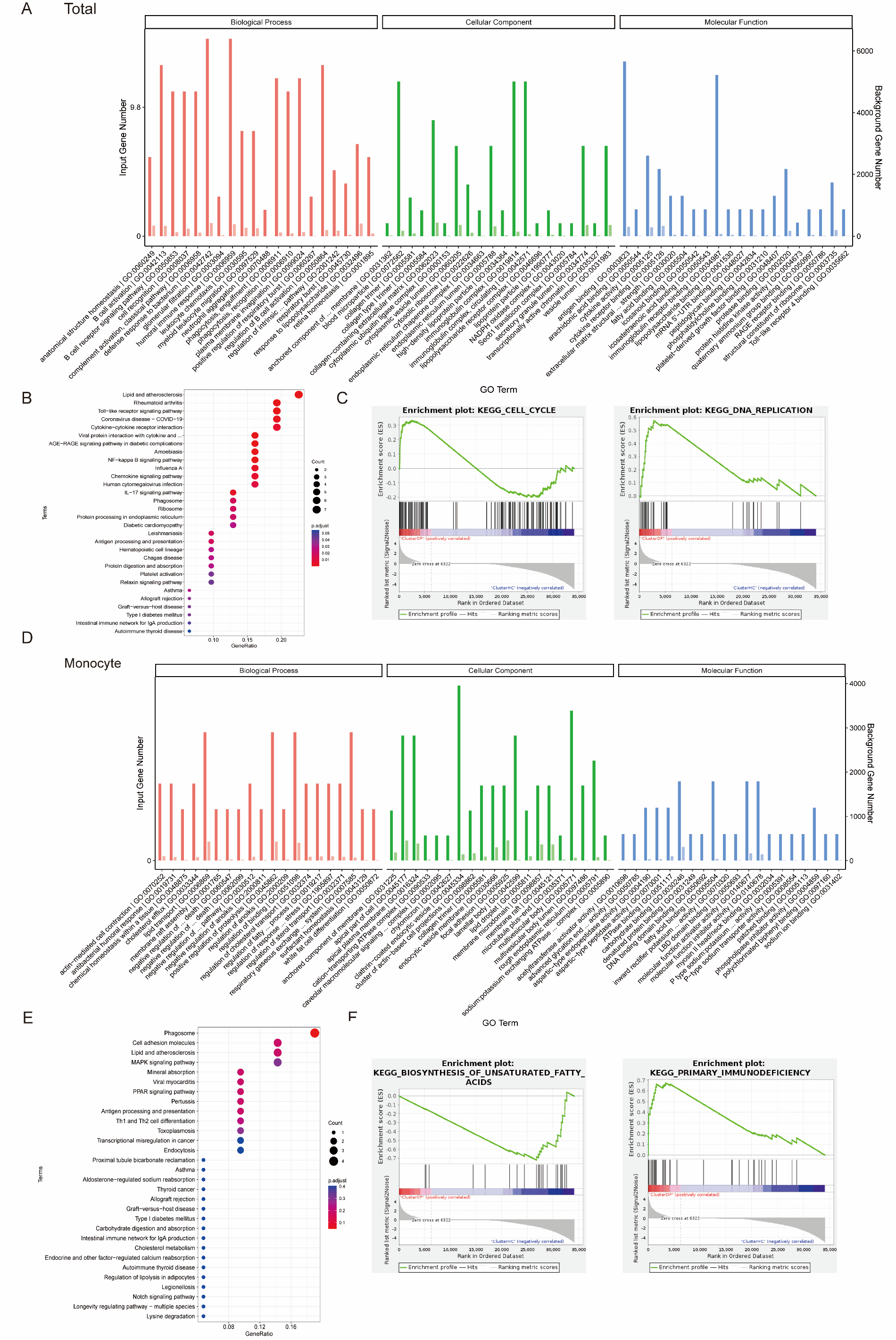


Figure S4. Functional analysis assessment in patients with OP. (A) GO enrichment analysis for the category of regulated genes between normal and OP patients. (B) KEGG analysis for the regulated pathways between normal and OP patients. (C) GSEA analysis revealed that the proteins were mainly enriched in cell cycle and DNA replication. (D) GO enrichment analysis for the category of regulated genes in monocytes between normal and OP patients. (E) KEGG analysis for the regulated pathways in monocytes between normal and OP patients. (F) GSEA analysis revealed that the proteins were mainly enriched in the biosynthesis of unsaturated fatty acids and primary immunodeficiency in monocytes of OP patients.

Figure S5


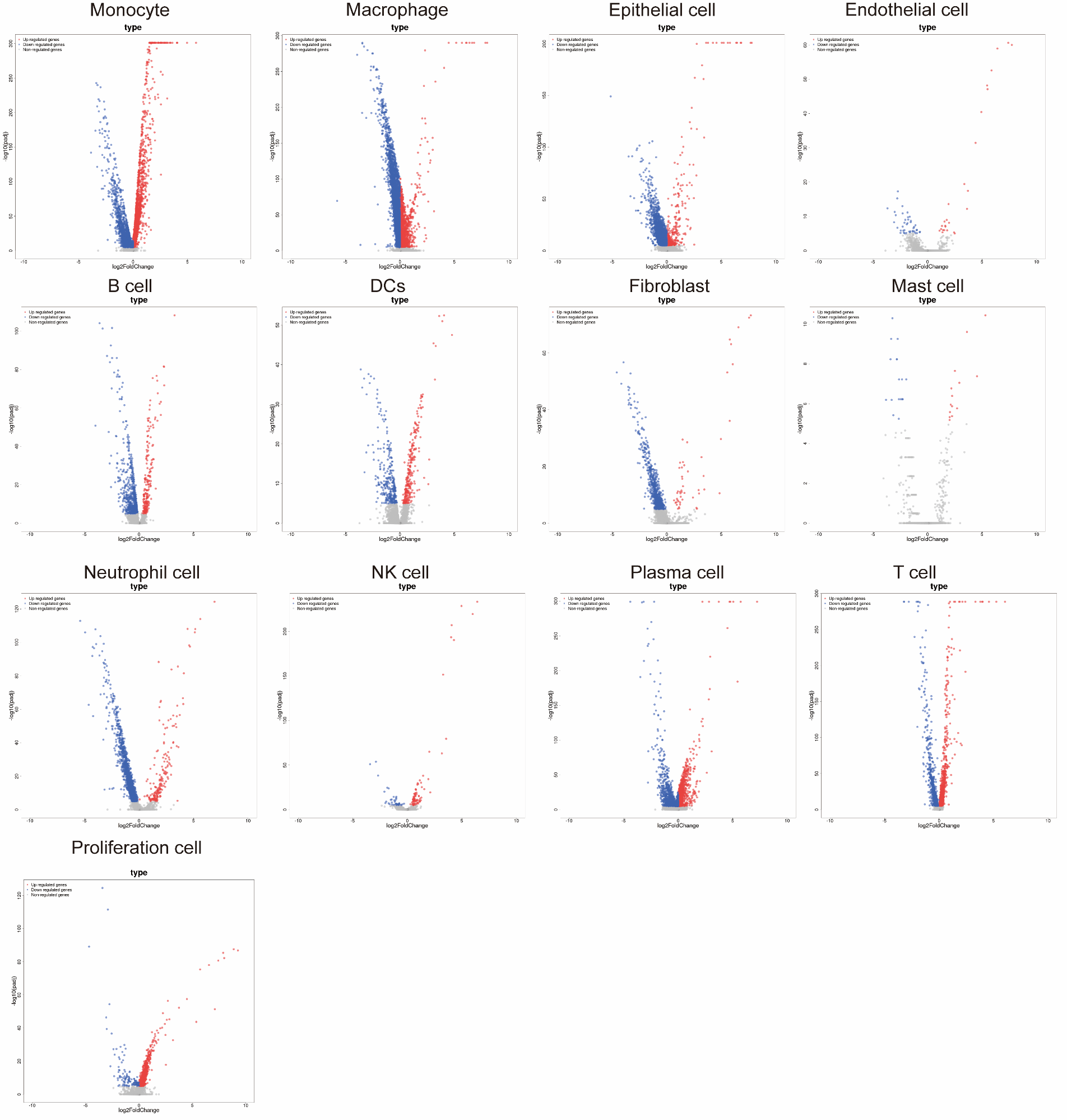


Figure S5. Volcano plots compare two groups as indicated in the plot. Groups with p-value <0.05 were considered significantly differential expressions. The scatter plot depicts the difference score of these terms versus the −log10-transformed p-value, which is shown with red (UP) and blue (DOWN).

Figure S6


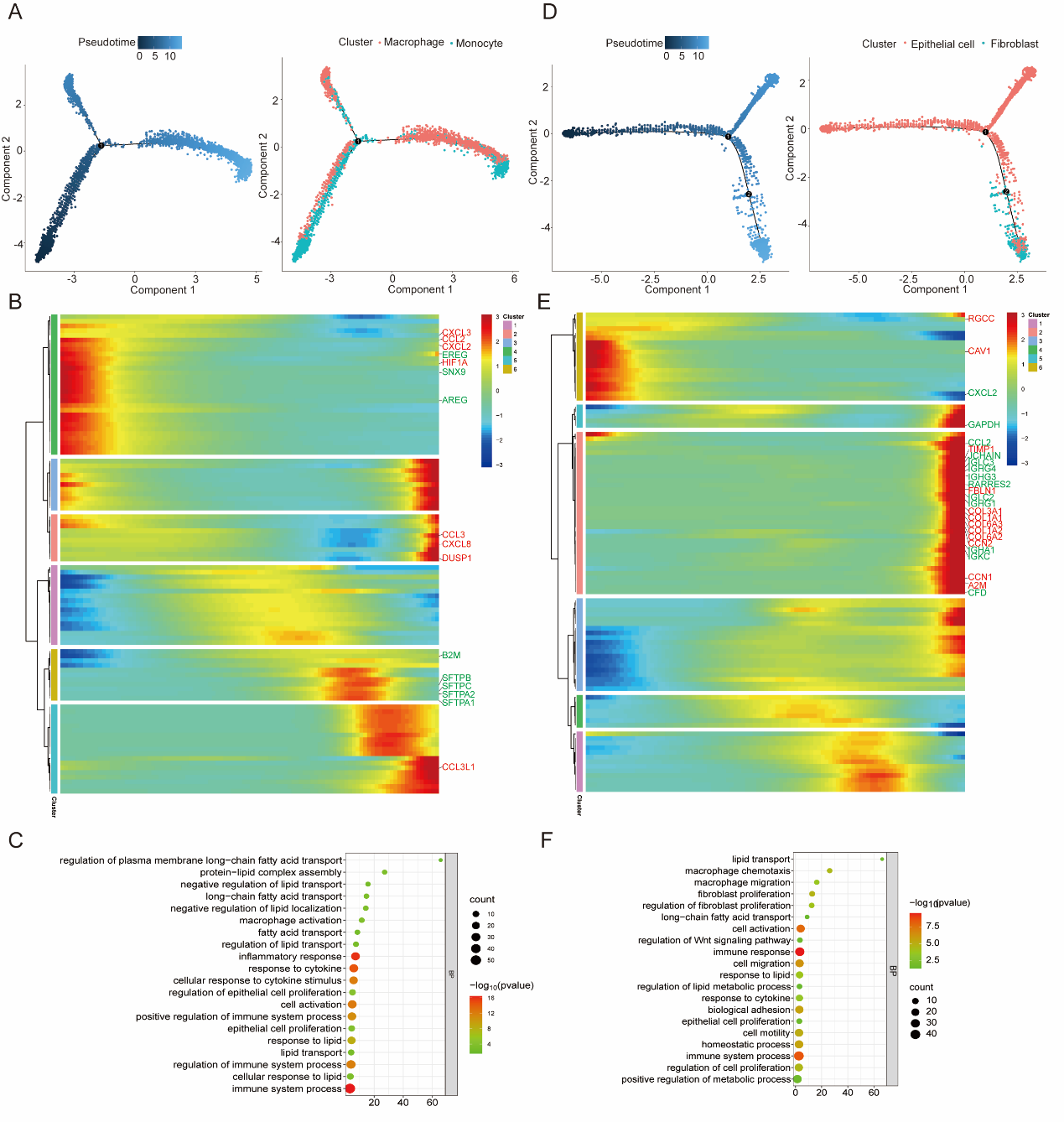


Figure S6. Pseudotime cell trajectory analysis on major cell types (monocyte-macrophage and epithelial cell-fibroblast). (A) Pseudo-time trajectory of monocyte-macrophage with gene expression profiles inferred by Monocle 2. The two primary components in this analysis are the horizontal and vertical coordinates. Each dot depicted in the figure corresponds to a cell, while the numbers enclosed within the black circle indicate the nodes responsible for determining distinct cell states in the trajectory analysis. In the left image, the gradient of colors from dark to light signifies the progression of pseudo-time, whereas the right image employs various colors to represent distinct cell clusters. (B) The differentially expressed genes along the pseudo-time (columns) were hierarchically clustered into two profiles of monocyte-macrophage. (C) BP of GO enrichment of scRNA-seq data in monocyte-macrophage of OP patients. (D) Pseudo-time trajectory of epithelial cell-fibroblast with gene expression profiles inferred by Monocle 2. (E) The differentially expressed genes along the pseudo-time were hierarchically clustered into two profiles of epithelial cell-fibroblast. (F) BP of GO enrichment of scRNA-seq data in epithelial cell-fibroblast of OP patients.

Figure S7


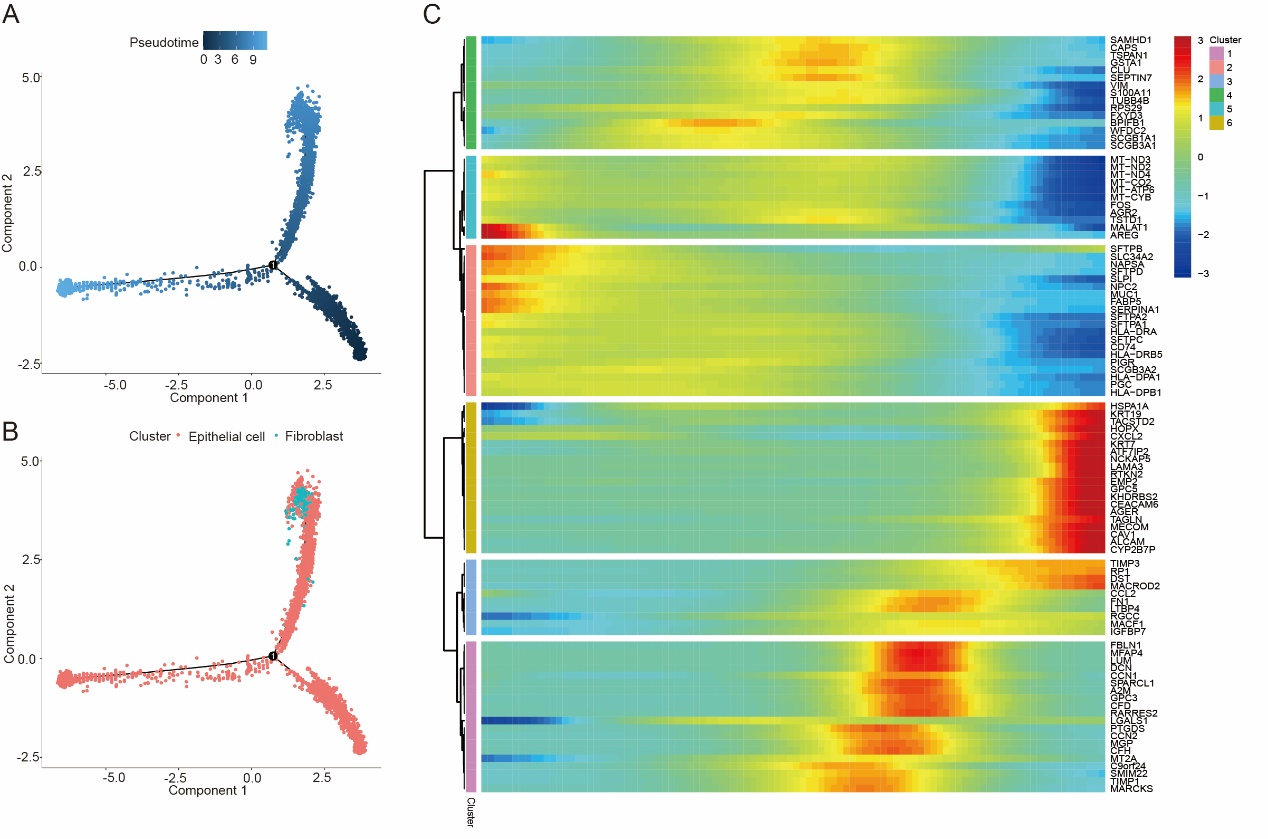
Figure S7. Pseudotime cell trajectory analysis on major cell types (epithelial cell-fibroblast in HC group). (A-B) Pseudo-time trajectory of epithelial cell-fibroblast of HC group with gene expression profiles inferred by Monocle 2. (C) The differentially expressed genes along the pseudo-time were hierarchically clustered into two profiles of epithelial cell-fibroblast in HC group.

Figure S8


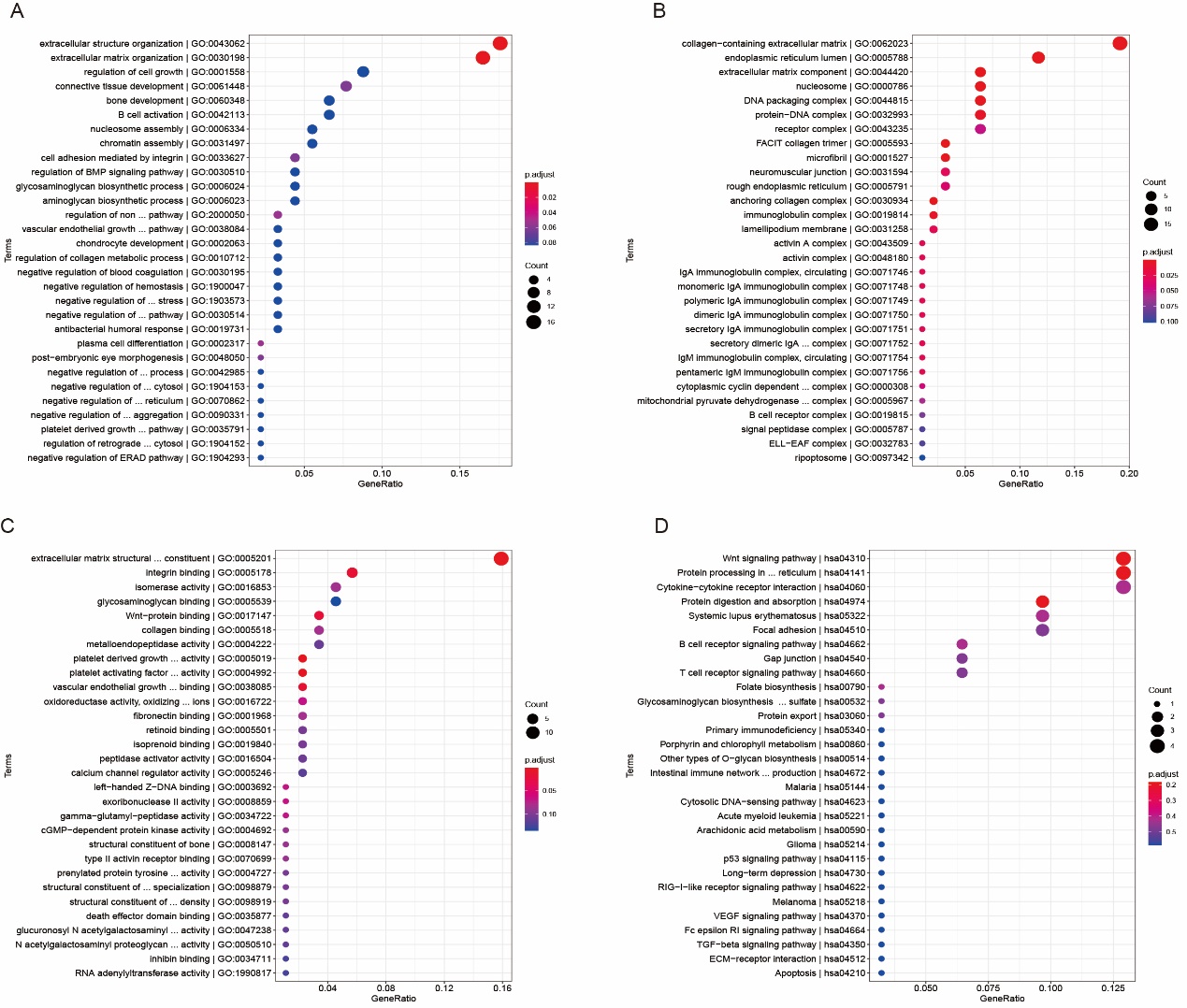


Figure S8. Functional analysis assessment with upregulated genes in patients with OP. (A-C) GO enrichment analysis (BP, CC, and MF) for the category of regulated genes between normal and OP patients. (B) KEGG analysis for the regulated pathways between normal and OP patients.

Figure S9


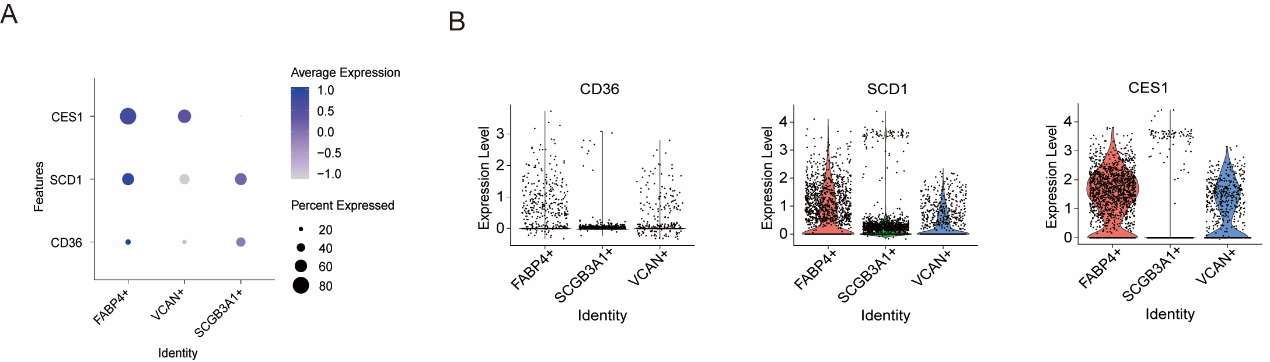


Figure S9. Single-cell sequencing was utilized to examine the expression levels of CD36, SEC1, and SCD1 across various subtypes of macrophages. (A) Dot plot showing the expression levels of CD36, SEC1, and SCD1 in the subclusters of macrophages (FABP4^+^, VCAN^+^, and SCGB3A1^+^). (B) Violin plot showing the expression levels of *CD36*, *CES1*, and *SCD1* in the three subclusters of macrophages.

Figure S10


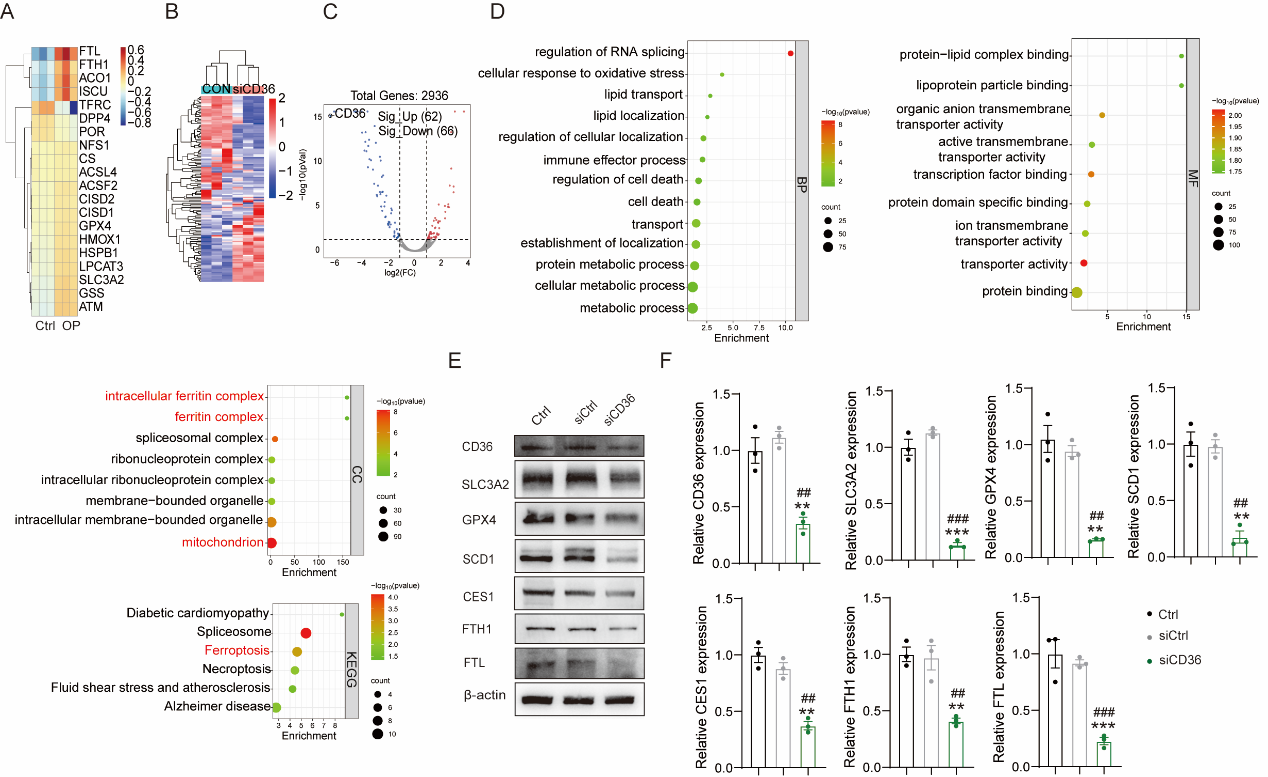


Figure S10. Reprogramming of lipid metabolism in alveolar macrophages promotes ferroptosis. (A) Heatmap showing the differentially expressed genes of ferroptosis in OP tissues by proteomics. (B) Unsupervised hierarchical clustering was performed on 242 LFQ-protein intensities (log2) that were quantified in three replicates of alveolar macrophages with knocking down CD36, identified by proteomics. (C) Distribution of proteins across two groups. Volcano plots of siCD36/CON protein comparisons. Proteins with *p* < 0.05 are present above the horizontal lines (grey). Proteins with significant fold-change (fold change > 2.0) are outside the grey vertical lines. The total number of proteins higher in expression (red circle) and lower in expression (blue circle) for each comparison was noted. (D) GO enrichment and KEGG analysis for the categories of up-regulated and down-regulated proteins between CON and siCD36 groups. (E-F) Western blot assay was used to detect the expression of CD36, SLC3A2, GPX4, FTH1, FTL, CES1, and SCD1 in alveolar macrophages.

Figure S11


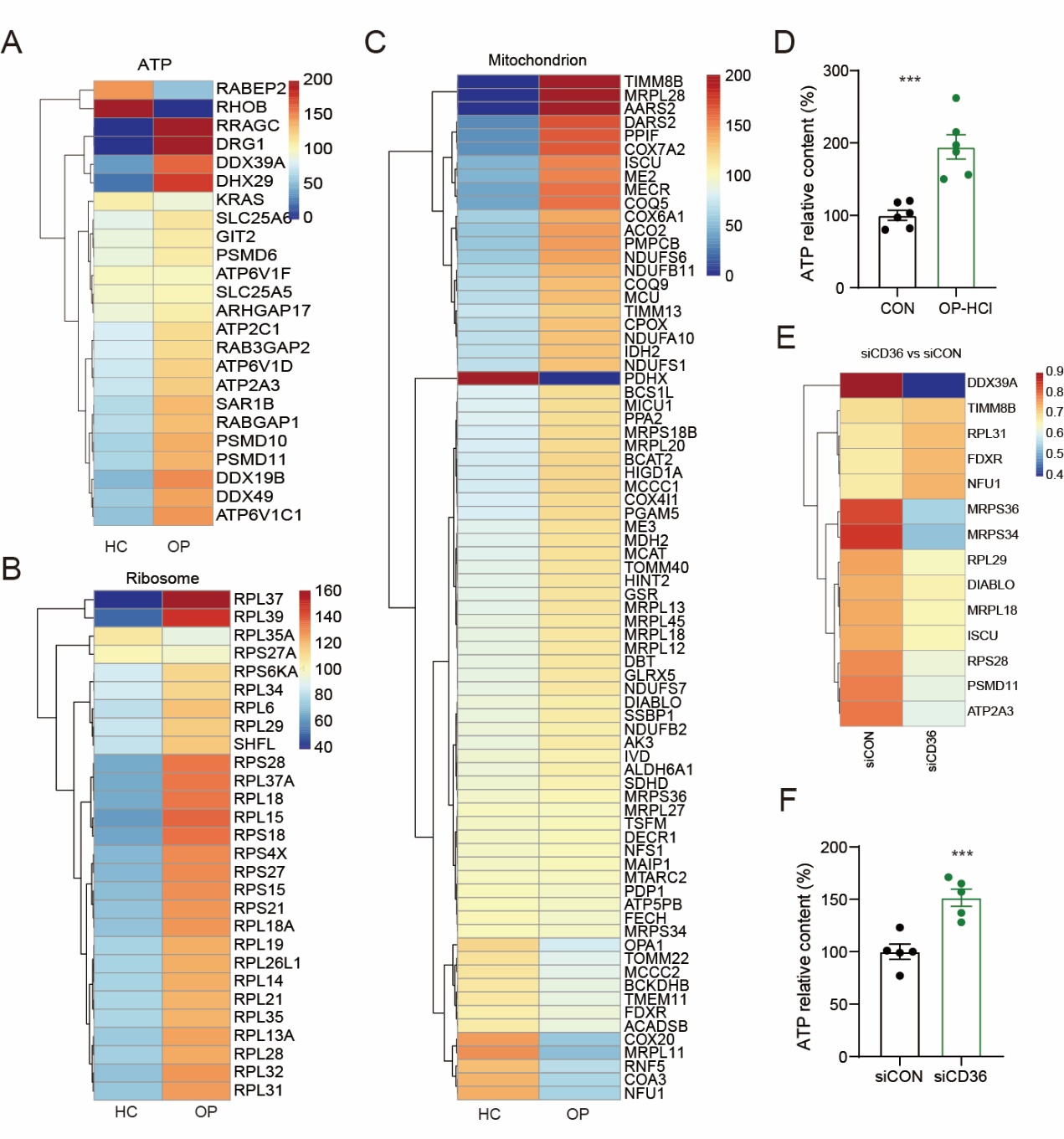


Figure S11. CD36 affects ATP and ribosomal components in macrophages. (A-C) The heatmap showed the differential expression of proteins related to energy and ribosome in OP patients compared with HC group. (D) Intracellular ATP measurement in OP mice model. (t-tests, *P<0.05, ***P<0.001) (E) The heatmap showed the differential expression of proteins related to energy and ribosome in siCD36 group. (F) Intracellular ATP measurement after 48 hours of siCD36 treatment. (t-tests, *P<0.05, ***P<0.001)

Figure S12


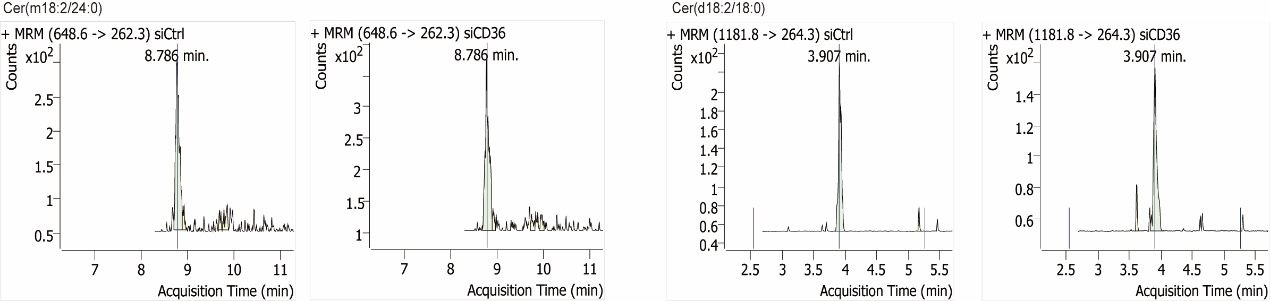


Figure S12. Effect of CD36 interference on the content of C16, C18, and C24 ceramides in mass spectrometry analysis.

Figure S13


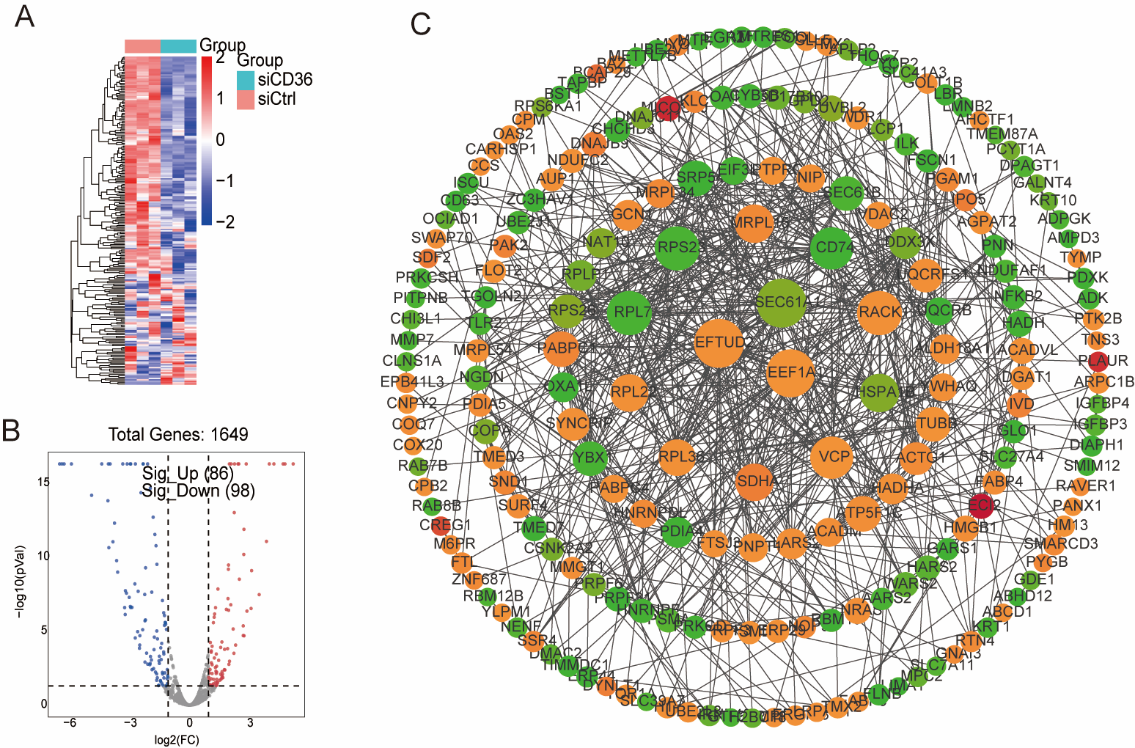


Figure S13. Proteomic analysis of alveolar macrophage secretions. (A) Heatmap showing the differentially expressed genes in the secretions of alveolar macrophages by knocking down CD36. (B) Distribution of secretions across two groups with volcano plots of siCD36/CON protein comparisons. (C) Network of differentially expressed secretions between CON and siCD36 groups.

Table S1

| Item | OP（43） | HEALTHY (57) | p |
| --- | --- | --- | --- |
| Gender |  |  |  |
| Male | 25 |  |  |
| Female | 18 |  |  |
| P_CO2_ | 36.43±2.31 | 40±2.55 |  |
| P_O2_ | 72.04±5.21 | 95.5±6.38 | ** |
| White blood cells | 8.56±3.56 | 6.5±1.53 |  |
| Percentage of neutrophils | 71.61±9.39 | 57.5±8.93 | * |
| Hemoglobin (male) | 136.00±12.53 | 152.5±11.48 | * |
| Hemoglobin (female) | 124.22±10.51 | 142.5±8.93 | * |
| Platelet count | 317.58±42.61 | 237.5±37.4 | ** |
| FIB | 4.47±0.4 | 3.68±0.66 | * |
| D-dimer | 1.31±0.55 | 0.25±0.13 | ** |
| Albumin | 33.84±5.02 | 42.5±3.83 | ** |
| GGT | 45.78±8.06 | 26±9.69 | ** |
| ESR (Erythrocyte Sedimentation Rate) | 34.89±8.96 | 10±5.1 | ** |
| CRP | 41.05±0.83 | 14.35±0.73 | ** |
| PCT | 0.81±2.6 | 0.047±0.02 | ** |

*p<0.05, **p<0.01

Table S2 Primer sequence for real-time polymerase chain reaction

| Names | Forward primer (5′ to 3′) | Reverse primer (5′ to 3′) | Amplified product  (bps) | Accession |
| --- | --- | --- | --- | --- |
| *Cd36* | CACAGCTGCCTTCTGAAATGTGTGG | TTTCTACGTGGCCCGGTTCTAATTC | 171 | NM_001159555.2 |
| *Scd1* | CGGTCATCCCATCGCCTGCTCT | GTAGGCGAGTGGCGGAACTGC | 133 | NM_009127.4 |
| *Ces1* | AGAGGAGCTCTTGGAGACGACAT | ACTCCTGCTTGTTAATTCC GACC | 97 | NM_021456.4 |
| *Gapdh* | TACGGGTGCACGTAGCTCA | GAGGGCTGCAGTCCGTATT | 128 | NM_008084.3 |
